# Vem++, a C++ library to handle and play with the virtual element method

**DOI:** 10.1007/s11075-025-02059-z

**Published:** 2025-03-29

**Authors:** Franco Dassi

**Affiliations:** Dipartimento di Matematica e Applicazioni, Università Milano - Bicocca, via Cozzi 55, Milano, 20126 Italy

**Keywords:** Virtual element method, Polytopal methods, C++

## Abstract

The Virtual Element Method (VEM) is an extension of the Finite Element Method (FEM) to handle polytopal meshes. After giving a short introduction of the VEM for a two dimensional Laplacian problem, we show the differences between an implementation of a VEM and a FEM code highlighting which are the main issues associated with the VEM framework. Furthermore, this paper will show one of the possible ways to face such issues: Vem++ a C++ library developed to “deal and play” with the VEM discretisation. This C++ library deals with the VEM, since there are several partial differential equations in two/three dimensions coming from both academic and engineering problems. Then, one can “play” with the VEM, since Vem++ has been designed so that one can plug-in new features such as new polytopes quadrature rules, new solvers and new virtual element spaces in a smart way.

## Introduction

The Virtual Element Method (VEM) was introduced for the first time in [1]. It is a generalization of the Finite Element Method (FEM) that provides some advantages. Fist, it allows the presence of general polytopal elements inside the mesh [2, 3], it can naturally handle hanging nodes so that refining processes and gluing pieces of meshes become more straightforward [4–7]. It ensures cheap mesh generation and matrix assembly in bulk-surface PDEs for both 2d and 3d case [8, 9]. Furthermore, since the VEM is an extension of the FEM, these two methods are perfectly compatible. Indeed, suppose to have a two dimensional mesh composed by triangles, squares and some polygons. Then, one can use the FEM spaces on triangles and squares while it can exploit the VEM spaces on polygons *without any* particular trick [7].

However, the advantages of the VEM are not limited to the meshing point of view. Indeed, the VEM spaces can be built in such a way that the discrete functions inherit some useful properties. In [10] the authors developed a family of VE spaces for the Stokes problem that are exactly “divergence-free” in contrast to the standard FEM approaches where such a property is obtained in a relaxed/weak sense, or using special discretisations, for instance the Scott-Vogelius type elements. Another example the flexibility of the VEM is given in [11]. In such work the VEM spaces are used to solve elasticity equations based on the Hellinger-Reissner variational principle. A standard FEM imposes the symmetry of the stress tensor variable in a weak sense. Such constraint increases the dimension of the linear system at hand and, consequently, the complexity in its resolution. The VEM overcomes this issue. Indeed, the symmetry of the tensor field is imposed within the discrete functional spaces. The result is a cheaper linear system to solve since the linear system does not have additional equations to impose the symmetry of the tensor field. Another advantage in modifying the VE spaces consists in curved domains. Specifically, it is possible to plug the curved geometry with-in the space [12–16].

Due to all these advantages, the VEM has reached a good level of success among researchers in different fields: solid mechanics [11, 13, 17, 18, 18], Maxwell equations [7, 19], wave equations [20] aeronautics [21, 22], morpho-chemical problems [23], reservoir simulations [24, 25] and discrete fractional network [26, 27].

Although the VEM is an extension of the FEM, it presents several challenges from the coding point of view. First, there is no a reference element since it is not apriori known the shape of the polygon we are considering. Basis functions are no more polynomials but virtual functions that are unknown and one does not need to compute them. Furthermore, since we are considering general polygons, one needs to find proper quadrature rules to compute integrals.

All these issues were faced in the C++ library Vem++. The aim of this paper is giving to the reader and future code developers a possible answer to each of these questions. Moreover, we will see that Vem++ was also designed to “play” with the VEM. Specifically, researchers can follow specific guidelines to plug in Vem++ their own polytopal quadrature rules, their own strategy to solve linear systems arising from the discretization of a partial differential equation or to make their VEM discretization of a specific problem. Such guidelines are briefly described in this paper, but there are also ten tutorials that help users to understand the philosophy of the code and, consequently, to develop what they need within Vem++.

Before going into the detail of the present paper, we mention that there are several VEM codes available. In [28], Sutton proposes an implementation of the VEM in *only* 50 lines of Matlab code for the two dimensional case. Another Matlab implementation of the high-order VEM is presented in [29]. In this case the aim of the authors is to furnish an easy-to-follow didactic implementation of the VEM in 2d to make a first step in the VEM framework. Another important code to mention is the one proposed by A. Russo during the Dobbiaco Summer School “Theory and Practice of the Virtual Element Methods” in 2018 freely available in [30]. Since also in this case the code was developed for didactic purposes, its implementation is only for the two dimensional case. Furthermore, another VE Matlab implementation is VEMcomp for bulk-surface PDEs in 2d and 3d [31]. Concerning C++ development of the VEM, we mention the following object oriented libraries VEMLab and Veamy [32, 33] both for the two dimensional case. Furthermore, there are ongoing work to have a VEM module in Deal II [34], while in Dune there is a module realizing the VEM in 2d where projections are built from constrained least squares problems [35, 36].

The paper is organised as follow. In Section 2 we will give a brief description of the VEM in a standard and easy-to-understand Poisson problem. Section 3 is the core of the paper: it contains a short description of the differences/analogies between a VEM and a FEM code. Then, Section 4 focuses on the description on how to plug in new features in Vem++. Finally, Section 5 describes the partial differential equations developed in Vem++ and more practical examples where such library was already applied. We underline that this last section will be not exhaustive since each topic requires its own paper to be better understood. However, for each example, we will give specific references so that the reader can have a more detailed description of them.

### Notation

Throughout this paper we will follow the usual notations for Sobolev and Hilbert spaces and norms [37]. We refer to polyhedrons as *P* and we denote their faces with *F*. The outward-pointing normal of the face *F* with respect to the polyhedron *P* is denoted by $\textbf{n}_F$. The volume of a polyhedron is |*P*|, its diameter is $h_P$ and the coordinates of its barycenter are $x_P$, $y_P$ and $z_P$. A generic two dimensional polygon is referred by *E* while *e* is always a generic edge. As for the polyhedron we refer to the area of a polygon *E* as |*E*|, $h_E$ is its diameter while the coordinates of its barycenter will be $x_E$ and $y_E$. We will use the $\partial$ operator to denote the boundary of a polygon or polyhedron. With some abuse of notation and to simplify the equations, we define $\partial P$ as the union of the faces of *P*, while $\partial E$ denotes the set of edges of a generic polygon *E*. Given a generic open domain $\mathcal {O}\subset \mathbb {R}^d$, with $d=1,2$ and 3, we refer to the polynomials of degree *k* defined on $\mathcal {O}$ as $\mathbb {P}_k(\mathcal {O})$. In these polynomial spaces, $\{m_\alpha \}$ will be the basis of the scaled monomials [38]. Moreover, a discrete function will be always referred with the subscript “*h*”, for instance $v_h$, and the basis functions coming from the virtual element approximation are always denoted by $\phi _i$.

## Discretization of a poisson problem in 2d

The Vem++ library can handle different types of both two and three dimensional PDEs, in Section 5 we will collect part of them. Here, we consider the Poisson equation in two dimensions, i.e.,

1 $$\begin{aligned} \left\{ \begin{array}{rll} -\Delta u=& f & \text {in}\,\Omega \\ u=& 0 & \text {on}\,\partial \Omega \end{array} \right. \,, \end{aligned}$$

where $\Omega$ is a domain in $\mathbb {R}^2$ and $f\in L^2(\Omega )$: such problem is easy, but it clearly shows which are the essential features of VEM so that the reader understand them and focus on their implementation.

In order to get the numerical approximation of the solution of problem (1), the virtual element method uses a standard Galerkin framework [39]: first, we introduce a continuous functional space, *V*, and define the variational formulation of (1). Now, the problem becomes:

*find* $u\in H^1(\Omega )$ *such that*

2 $$\begin{aligned} \int _\Omega \nabla u \cdot \nabla v ~\text {d}\Omega = \int _\Omega f v ~\text {d}\Omega \qquad \forall v\in H^1_0(\Omega )\,. \end{aligned}$$

Then, we discretise, $\Omega _h$, and define on such a mesh a discrete space $V_h\subset V:=H_0^1(\Omega )$. We approximate the continuous bilinear forms in (2) and split them among mesh elements, i.e.,

3 $$\begin{aligned} a(\cdot ,\cdot )\approx a_h(\cdot ,\cdot )=\sum _{E\in \Omega _h} a_h^E(\cdot ,\cdot )\,, \end{aligned}$$

where *E* is a generic element of the mesh $\Omega _h$. Concerning the right hand side, we introduce the discrete function $f_h$ that is an approximation of the load term *f*.

In the following paragraphs we will give a brief description on how the VEM creates the discrete space $V_h$ and the bilinear forms, we refer the reader to [1, 38] for a deeper analysis.

### Discrete space $V_h$

In the FEM, the discrete space $V_h$ is split over the mesh elements and such a space restricted on a mesh element is a polynomial of a specific degree. In the VEM, $V_h$ is still split over mesh elements, but its restriction on an element is no more a polynomial, it is an unknown function $v_h$ defined as

4 $$\begin{aligned} V_h(E) :=\left\{ v_h\in H^1(E)\cap C^0(\partial E)\,:\, \Delta v_h\in \mathbb {P}_{k-2}(E), \quad v_h|_e\in \mathbb {P}_k(e)\,\forall e\in \partial E\right\} \,. \end{aligned}$$

However, if we look at the definition of $V_h(E)$, we observe that this space still contains polynomials of degree *k*, but it is enriched by other unknown functions such that their Laplacian is a polynomial of degree $k-2$ and that are polynomials of degree *k* on $\partial E$. The presence of such unknown (virtual) functions is the key point of the VEM. Specifically, their presence makes possible to handle arbitrarily shaped polygons also characterised by hanging nodes in a smart way.

Since basis functions in a FEM framework are polynomials of a given degree, we can use point-wise evaluations to uniquely determine them. In the VEM, basis functions are solutions of a PDE, c.f. (4), so we consider a more general concept of degrees of freedom. Indeed, the degrees of freedom are functionals that uniquely determine the function $v_h$ in $V_h(E)$.

In this specific case, a function $v_h\in V_h(E)$ is uniquely determined by the following degrees of freedom:

D1: the value of $v_h$ at the vertexes of *E*;
D2: the value of $v_h$ at $k-1$ points inside each edge $e\in \partial E$;
D3: the moments of $v_h$ inside the polygon *E*
  5 $$\begin{aligned} \frac{1}{|E|}\int _E v_h\,m_\alpha ~\text {d}E\,. \end{aligned}$$

**Fig. 1:**
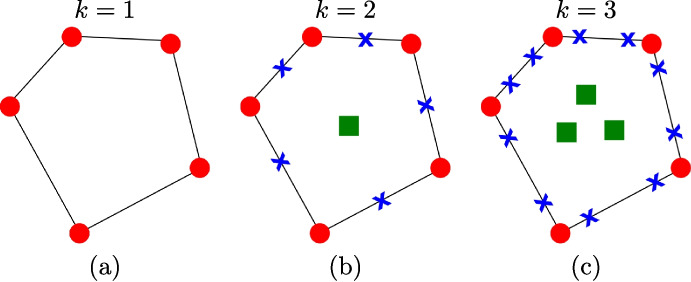
An example of a polygon where we highlight the dof for a VEM approximation of degree $k=1,2$ and 3

In Fig. 1, we show the degrees of freedom for a function $v_h\in V_h(E)$ with different values of *k*. For $k=1$ the virtual functions are harmonic and polynomials of degree 1 over $\partial E$. As a consequence the function $v_h$ is uniquely determined by the values of $v_h$ at the vertexes of *E*, see the red bullets in Fig. 1(a).

For $k=2$, $v_h$ is a polynomial of degree 2 over edges and its laplacian is a constant polynomial. Then, to uniquely determine $v_h$, we need 3 points on each edge: the values of $v_h$ at the edge endpoints (red bullets in Fig. 1(b)), and the value of $v_h$ at the edge midpoints (the blue crosses Fig. 1(b)). Finally, we need one internal moment to fix the constant Laplacian (the green square in Fig. 1(b)).

Then, in Fig. 1(c) we show the degrees of freedom for the case $k=3$. Since $v_h$ is a polynomial of degree 3 over each edge of *E*, four points evaluations are needed (two red bullets and two blue crosses). The, since the Laplacian of $v_h$ is now a polynomial of degree 1, we need three moments (green squares in Fig. 1(c)).

#### Remark 2.1

As for the FEM, in the virtual element setting a basis function is associated with a specific degree of freedom, i.e., there is one specific degree of freedom that is one and all the other ones are zero. However, since D1, D2 and D3 are not only point-wise evaluations, the virtual basis functions have specific characteristics according to them. Consider, for instance, a pentagon and a VEM approximation degree $k=3$. The basis function $\phi _A$ that is associated with *A*, has the following characteristics:

- it is zero in all the degrees of freedom D1 but the one assoicated with *A*;
- it is zero in all the degrees of freedom D2;
- the following relation holds
  $$\begin{aligned} \frac{1}{|E|}\int _E \phi _A ~\text {d}E= &   0, \\ \frac{1}{|E|}\int _E \phi _A \left( \frac{x-x_E}{h_E}\right) ~\text {d}E= 0\,&\quad \text {and}\quad&\frac{1}{|E|}\int _E \phi _A \left( \frac{y-y_E}{h_E}\right) ~\text {d}E= 0. \end{aligned}$$

Then, if we are considering a basis function $\phi _{\mathcal {M}}$ which is associated with the “*x* moment”, it has the following properties:

- it is zero in all the degrees of freedom D1;
- it is zero in all the degrees of freedom D2;
- the following relation holds
  $$\begin{aligned} \frac{1}{|E|}\int _E \phi _{\mathcal {M}} ~\text {d}E= &   0, \\ \frac{1}{|E|}\int _E \phi _{\mathcal {M}} \left( \frac{x-x_E}{h_E}\right) ~\text {d}E= 1\,&\quad \text {and}\quad&\frac{1}{|E|}\int _E \phi _{\mathcal {M}} \left( \frac{y-y_E}{h_E}\right) ~\text {d}E= 0. \end{aligned}$$

**Fig. 2:**
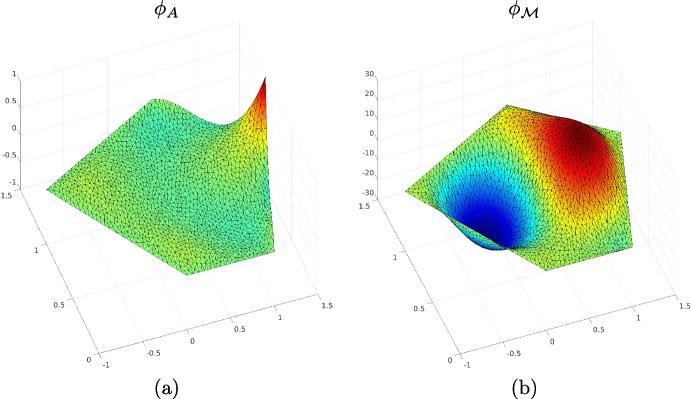
The shapes of the basis functions $\phi _A$ and $\phi _{\mathcal {M}}$

Going against the “VEM philosophy” we compute both $\phi _A$ and $\phi _{\mathcal {M}}$ and we show their shape in Fig. 2(a) and (b), respectively. To achieve this goal, we use a FEM code and solve a proper PDE.

In the following sections, we see that although we do not have an explicit expression of the functions in $V_h(E)$, once we know their degrees of freedom, we are able to set a discretisation of the problem defined in (2) and get the solution.

**Fig. 3:**
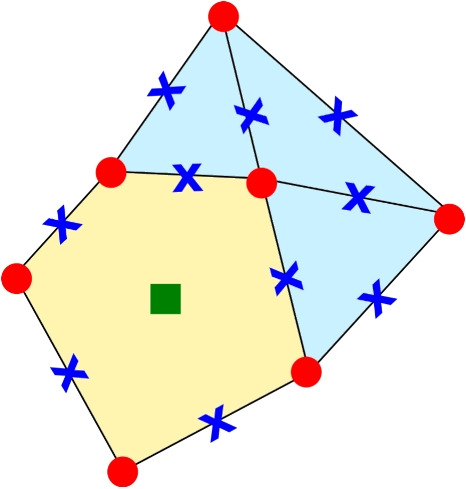
Merging between a VEM (light yellow) and FEM (light blue) elements when we are considering an approximation degree $k=2$. We further underline that the compatibility is possible *for each* degree *k*

Before going into the detail on how define the bi-linear forms, we further underline an important aspect of the VEM. From the definition of $V_h(E)$ and its degrees of freedom, we observe that a function $v_h\in V_h(E)$ is a polynomial of degree *k* on $\partial E$ and its degrees of freedom coincide with the FEM ones on edges. This aspect is a key point that allows us to combine FEM and VEM together. Specifically, in a PDE discretisation one can use the VEM only when the elements are polygons with more than four edges and the FEM on all triangular or quadrilateral elements, see Fig. 3.

### Projector operator

In this section we will introduce the projection operator $\Pi ^\nabla _{k}$. We will see that such projection operator is the key ingredient to construct the bi-linear operator $a_h^E(\cdot ,\cdot )$. Given a function $v_h\in V_h(E)$, we define the projection $\Pi ^\nabla _{k}: V_h(E)\rightarrow \mathbb {P}_k(E)$ as

6 $$\begin{aligned} \left\{ \begin{array}{rcl} \displaystyle {\int _E} \nabla \Pi ^\nabla _{k}v_h\cdot \nabla p_k~\text {d}E& =&  \displaystyle {\int _E} \nabla v_h\cdot \nabla p_k~\text {d}E\qquad \forall p_k\in \mathbb {P}_k(E) \\[1em] \displaystyle {\int _{\partial E}} \Pi ^\nabla _{k}v_h ~\text {d}e& =&  \displaystyle {\int _{\partial E}}v_h ~\text {d}e\end{array} \right. \,. \end{aligned}$$

The projection $\Pi ^\nabla _{k} v_h$ is a polynomial of degree *k* and it can be computed via the degrees of freedom of $v_h$, i.e., we can compute $\Pi ^\nabla _{k}v_h$ *without* knowing $v_h$ itself. This fact is well firmed in the VEM literature, see for instance [1, 38], and we will describe how it is computed later in Section 3.1 where we also show how such computation is handled in Vem++.

We can also define an $L^2$ projection operator $\Pi ^0_{k-2}$ for $k\ge 2$ which will play a crucial role in approximating the right-hand side of (1). Given a function $v_h\in V_h(E)$, we define the $L^2$ projection operator $\Pi ^0_{k-2}:V_h(E)\rightarrow \mathbb {P}_{k-2}(E)$ as

7 $$\begin{aligned} \int _E \Pi ^0_{k-2}v_h p_{k-2}~\text {d}E&= \int _E v_h p_{k-2}~\text {d}E\qquad \forall p_{k-2}\in \mathbb {P}_{k-2}(E). \end{aligned}$$

Such a projection operator is computable. Indeed, if $k\ge 2$ the right-hand sides of (7) are exactly the degrees of freedom of $v_h$ so we know their values, see Fig. 1(b) and (c). However, if $k=1$, we know only the values of the virtual function $v_h$ at the vertexes of the polygon, see Fig. 1(a), so we can not compute the $L^2$ projection operator defined in (7). As a consequence, in the lowest order case, we define the following projection operator on constants

8 $$\begin{aligned} \Pi ^0_{0}v_h&= \frac{1}{N_V}\sum _{i=1}^{N_V} v_h(V_i)\,, \end{aligned}$$

where $N_V$ is the number of vertexes of *E* and $V_i$ is the $i-$th vertex of the polygon *E*.

#### Remark 2.2

If we properly change the virtual element spaces (the so-called *enhancing procedure*), it is possible to define an $L^2$ projector onto $\mathbb {P}_{k}$ instead of $\,\mathbb {P}_{k-2}$, which can be useful to get a better approximation of the right hand side term and to construct the mass term. From a theoretical point of view such procedure is advanced and it can not be explained in few words. However, from the coding point of view, once you understand how to build the $\Pi ^\nabla _{k}$ projection in this basic setting, it is clear how to construct the aforementioned $\Pi _k^0$ [38].

#### Remark 2.3

The VEM always involves the use of a problem-specific projection operator. For example, in the discretisation of a Stokes problem, a suitable $L^2$ projection operator $\boldsymbol{\Pi }^0_k$ is defined to approximate the mass operator [21]. On the other hand, for an elasticity problem, a different projection operator $\boldsymbol{\Pi }^\epsilon _k$ is employed to obtain the virtual element approximation of the symmetric gradient [21]. However, in all these cases, the projection operator can be computed solely based on the degrees of freedom, without requiring an explicit expression of the virtual function $v_h$.

### Problem forms

After introducing the projection operator we are ready to give the VEM approximation of both the local bilinear form $a_h^E(\cdot ,\cdot )$, (3), and the right hand side. Let us consider the projection operator $\Pi ^\nabla _{k}\phi _i$ of a generic virtual element basis function. We apply the continuous local form $a^E(\cdot ,\cdot )$ to

$$ \Pi ^\nabla _{k}\phi _i + \phi _i - \Pi ^\nabla _{k}\phi _i\,, $$

then, using the bi-linearity of such form and the orthogonality property in the definition of the $\Pi ^\nabla _{k}$ projection operator (the first condition of the linear system in (6)) we get the form

$$ a\left( \Pi ^\nabla _{k}\phi _i,\,\Pi ^\nabla _{k}\phi _j\right) + a\left( \phi _i -\Pi ^\nabla _{k}\phi _i,\,\phi _j-\Pi ^\nabla _{k}\phi _j\right) \,. $$

Since we are able to compute the projection $\Pi ^\nabla _{k}$, we can handle the first term, while the second one still contains the virtual function so it can not be computed. However, such term can be replaced with *any* positive definite bi-linear form $s^E(\cdot ,\,\cdot )$ computable form the degrees of freedom that satisfies for each $v_h\in V_h\cap \ker (\Pi ^\nabla _{k})$

$$ \alpha _*\,a^E(v_h,\,v_h)\le s^E(v_h,\,v_h) \le \alpha ^*\,a^E(v_h,\,v_h)\,, $$

where $\alpha _*$ and $\alpha ^*$ are positive constants. In the virtual element setting, there are several choices of such form and *all* of them can be computed although we are considering a virtual function $v_h$. Then, starting from this consideration we are now ready to set the local discrete form

9 $$\begin{aligned} a_h^E(\phi _i,\,\phi _j):= a\left( \Pi ^\nabla _{k}\phi _i,\,\Pi ^\nabla _{k}\phi _j\right) + s^E\left( \phi _i -\Pi ^\nabla _{k}\phi _i,\,\phi _j-\Pi ^\nabla _{k}\phi _j\right) \,. \end{aligned}$$

The fist part of this form is commonly called *consistency* and it ensures that the present method is exact when the solution is a polynomial. While the second part is called *stability* and it guarantees the right convergence rate of the solution.

In order to get a suitable approximation of the right hand side, we use the $\Pi _{k-2}^0$ projection operator. Since the definition of such operator depends on the degree *k*, we distinguish two cases:

- if $k=1$ we approximate the integral using the projection defined in (8) on both *f* and $\phi _i$:
  $$ \int _E \Pi _{0}^0\,f \,\Pi _{0}^0\phi _i~\text {d}E= \Pi _{0}^0\,f \,\Pi _{0}^0\phi _i |E|\,, $$
- if $k\ge 2$ we apply the $L^2$ projection defined in (7) on the load term *f*, $f_h:=\Pi _{k-2}^0\,f$ and we proceed as follows exploiting the properties of this projection operator:
  $$ \int _E \Pi _{k-2}^0\,f \,\phi _i~\text {d}E= \int _E \Pi _{k-2}^0\,f\, \Pi _{k-2}^0 \phi _i~\text {d}E= \int _E f\, \Pi _{k-2}^0 \phi _i~\text {d}E\,. $$

Now we have all the ingredients to assemble the linear system associated with the virtual element approximation of the problem defined in (1). The theory behind the construction of the linear form $a_h$ and the right hand side is well-assessed in the literature, we refer the reader to [1] for the theoretical analysis of them, and to [38] for a more practical “guide” on their construction. Before starting the next section, we would like to underline that such assembling process is similar to the followed by the FEM. In both cases we have local basis functions, $\phi _i$, that defines *local* linear and bilinear forms. Then, they are spread in the global matrix. Finally, the solution is obtained via the resolution of a linear system. In Section 3.2 we will go more into the detail about such procedure, but for the moment it is easily understandable that the VEM and the FEM follow a similar workflow to get the discrete solution of a PDE.

## Implementation details

In this section we analyse the VEM from a more practical point of view. In Section 3.1, we describe the main difficulties in coding the VEM with respect to the FEM. Then, in Section 3.2 we underline the analogies between these two methods. In both sections we also provide some details on the structure of the Vem++ library.

### Differences with the FEM

In this subsection, we will describe the main difficulties in coding the VEM. Specifically, in Section 3.1.1 we focus on the difficulties related to the presence of arbitrarily shaped polygons. Then, in Section 3.1.2, we discuss how in Vem++ virtual basis functions are handled. Finally, in Section 3.1.3, we consider the problem of making integrals over polygons/polyhedrons and how to deal with the integration of virtual functions.

#### Element shape

The VEM can handle a mesh composed by arbitrarily shaped polygons and polyhedrons. As a consequence, since we do not have any reference shape, we need to take into account all possible cases. For instance, given a generic polygon, we can not a priori say that it has a fixed number of edges or vertices. Moreover, due to the flexibility of VEM, we can not either say that it has no hole! As a consequence, in Vem++ polygons and polyhedrons are managed as 2d and 3d *Piece-wise Linear Complex* (PLC), respectively [40]. The PLC is a typical structure used as input for the most common mesh generation software, see, e.g., triangle [41] and tetgen [42].

***Facet***

**Fig. 4:**
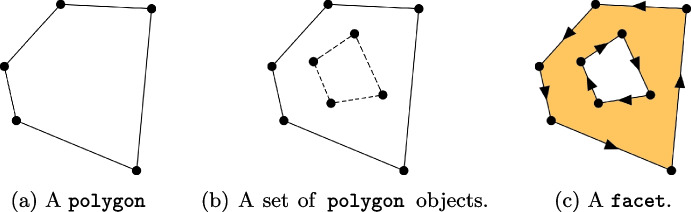
The data structure for a 2d PLC used in Vem++

The facet is the topological object used in Vem++ to handle a generic 2d PLC. It is composed by a collection of polygon, i.e., a sequence of points that identify a close piece-wise linear complex, see Fig. 4(a). Among this collection, there is the main polygon that encloses all the other ones, see Fig. 4(b). Then, the generic facet is defined running counter-clock-wise the main polygon and clock-wise the other polygons which define Facet’s holes, see Fig. 4(c).

As for the standard FEM, in some cases a virtual element approximation requires the definition of the out-ward pointing normal. However, thanks to the proposed orientation criterion, it is always possible to define out-pointing normal of each facet’s edge although it has holes. Moreover, one can exploit this orientation to compute the area in a straightforward way using the so-called shoelace formula [43].

##### Coding hint 3.1

The area of a generic facet is given by the main polygon area minus the area of all holes.

***Polyhedron***

**Fig. 5:**
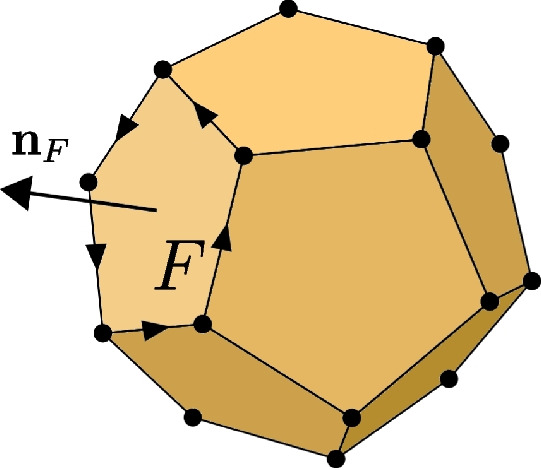
An example of polyhedron in Vem++ where we highlight the orientation of one facet so that the normal points outside

To handle a polyhedron in Vem++, we use a generic 3d PLC data structure. Also in this case we can not make any a priori assumption on the shape of the element: and it can also have holes inside. As a consequence, a polyhedron is handled by a C++ class called polyhedron that is a collection of facet and, eventually, a sequence of points that identify the interior of each hole. In both 2d and 3d mesh generation software, see for instance [41, 42], this is a common way to identify holes in the input domain. The facet are oriented in such a way that normal constructed via the right-hand rule points outside the polyhedron, see Fig. 5.

This kind of orientation plays a key role in the computation of the volume enclosed by the polyhedron. Indeed, one can use the divergence theorem to exactly compute the volume from face integrals. Consider a polyhedron *P* and a vector field $\textbf{F}$ such that its divergence is 1. Then, the following relation holds

10 $$\begin{aligned} |P| = \int _P ~\text {d}P= \int _P \text {div}(\textbf{F})~\text {d}P= \sum _{F\in \partial P}\int _{F} \textbf{n}_F\cdot \textbf{F}~\text {d}F\,. \end{aligned}$$

##### Coding hint 3.2

As vector field $\textbf{F}$ in (10), one can take $\textbf{F}(x,\,y,\,z)=(x,\,0,\,0)^\top$. Such choice is particularly appealing from the computational point of view. Indeed, the vector field we are integrating over faces is a polynomial of degree 1 and consequently we may use a low order quadrature rule to “exaclty” compute the face integral with the smallest computational effort.

**Fig. 6:**
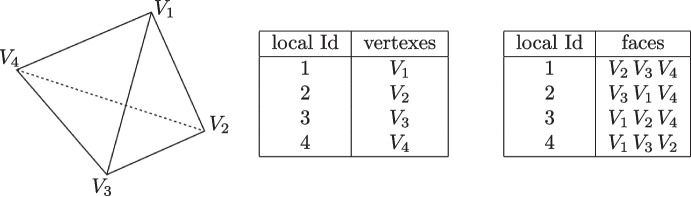
A tetrahedron with an example of a FEM data structure for its vertexes and faces. Notice that the local identifiers of the faces correspond to the identifier of the opposite vertex

It is worth mentioning the following coding aspect. In the most common FEM code vertexes, edges and faces have a specific order inside the data structure of an element [42, 44]. Consider, for instance, the faces stored inside the data structure of a tetrahedron. They are usually stored the following way: the first local face is the one opposite to the first vertex stored, the second one is opposite to the second vertex and so on, see Fig. 6.

Since the VEM can handle generic polyhedrons and finding a specific rule to store vertexes, edges and faces of an arbitrary shaped polygons is not straightforward, in Vem++ vertexes, edges and faces are randomly stored inside the data structure of a polyhedron.

#### Definition of the degrees of freedom

In a virtual element framework basis functions are not polynomials as for the FEM, see Section 2.1. This is the key feature of the VEM: the basis functions are virtual, we do not know them or, it will be better saying, we *do not want* to have their explicit expression. Indeed, to solve a partial differential equation via the VEM, the *only* information we need are the values of virtual basis functions on degrees of freedom and their projection operators.

The structure of Vem++ follows the same philosophy: there is no an explicit expression of the virtual basis functions, but they are a collection of information stored in the class vemDofDescription.

To better understand this fact, we consider the degrees of freedom described in Fig. 1(c), i.e., a VEM approximation degree $k=3$, and we show how the class vemDofDescription describes them in Vem++.

First, each dof has a tag so that we can recognise among each type. For instance, in this case we can distinguish among red bullets, blue crosses and green squares. Then, according to the tag, the class vemDofDescription stores the main information to deal with the dof:

- for red bullets it contains the identifier of the vertex the dof is associated with and the coordinates of such point;
- for each blue crosses, it contains the identifier of the edge the dof belongs to and the coordinate of the point;
- for each green square the class vemDofDescription contains the identifier of the face the dofs belongs to and the exponents of the monomial it is associated with. Notice that for this simple case we have three internal moments. Then, the set of exponents are the couples (0,0), (1,0) and (0,1) that represent the following monomial moments:
  $$ \int _E \phi _i~\text {d}E,\qquad \int _E \phi _i\left( \frac{x-x_E}{h_E}\right) ~\text {d}E\quad \text {and}\quad \int _E \phi _i\left( \frac{y-y_E}{h_E}\right) ~\text {d}E\,, $$
  respectively.

##### Remark 3.1

The information on the degrees of freedom are related to the virtual element space we are considering. If we consider a different virtual element space, the information stored inside vemDofDescription change. For instance, if you are considering the VEM spaces used to solve a Stokes problem we have moments associated with the divergence of the virtual basis function [21, 45].

#### Integration

As for the FEM, also in the VEM one needs to compute integrals. However, in this framework there are two main issues. On the one hand, we have to integrate over polygons. On the other hand, we have to integrate virtual basis function whose value can be unknown at quadrature points.

#### Integration over polygons

There are different ways to achieve this goal. The more straightforward one is to sub-triangulate polygons and use triangle quadrature points and weights. A more sophisticated strategy is to use quadrature rules built ad-hoc for general polygons/polyhedrons. Among them we mention Vianello formulas for two dimensional polygons [46], that was extended for polyhedrons in [16], and Chin-Sukumar quadrature rule [47]. In the majority of the cases, such rules result in a collection of more than needed quadrature points and, as a consequence, a high computational effort.

**Fig. 7:**
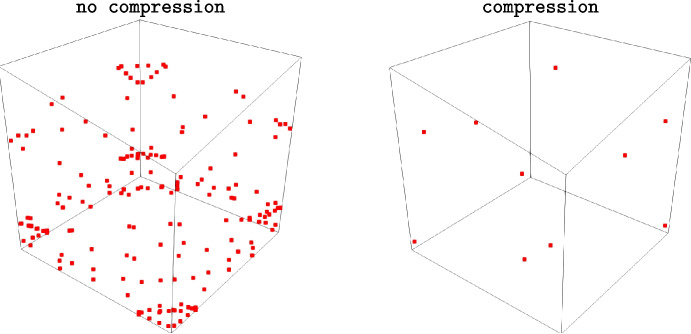
Number of quadrature points without and with the compression procedure. In the former case we have 192 points, while in the latter only 10 points

To avoid this issue, one possible strategy is to use compression rules that are able to reduce the number of points according to the quadrature rule precision required [48]. Such compression procedure is general and it is based on the resolution of a non-negative least squares problem that also ensures non-negativity of weights. To show the effectiveness of the compression procedure, we consider a cube and the quadrature formula that exactly integrates polynomials of degree 2 [16]. A standard rule has 192 quadrature points while the compressed one has only 10 points, see Fig. 7.

Depending on the shape of the polyhedron, this compression strategy may yield the minimum number of points required to interpolate a specific polynomial within this specific polyhedron. In this particular case, we consider a quadrature rule of degree 2. As a result, the compression procedure selects and adjusts the weights of 10 quadrature points, which is exactly the number needed to construct a degree-2 polynomial in three variables. However, when the polyhedron has a less regular shape, the efficiency of this compression decreases, leading to a higher number of quadrature points.

#### Integration of virtual basis functions

An integration quadrature rule is generally based on weighs and function evaluations. As a consequence, since in the VEM we do not have the explicit expression of the virtual basis functions, one may think that it is impossible to compute integrals that include a virtual function. However, this statement is not completely true. Indeed, it is possible to accurately compute *all* the integrals required to assemble the linear system and get the discrete solution and such computations are exact and fast.

In the following paragraphs, we will give a better explanation of this fact considering three issues: integral on edges, on faces and computing the integrals required to get the $~\Pi ^\nabla _{k}$-projection.

***Edge integrals and virtual functions***

Suppose that you want to compute the following integral

11 $$\begin{aligned} {\int _e v_h\,g ~\text {d}e\,,} \end{aligned}$$

where $v_h$ is a virtual function and *g* is a generic function. In the virtual element setting described in Section 2, $v_h$ is a polynomial of degree *k* on the boundary that can be reconstructed starting from the degrees of freedom of $v_h$ on the edge *e*. As a consequence, it is possible to compute the integral in (11) and its value is accurate according to the quadrature rule taken into account.

Furthermore, if $g\in \mathbb {P}_s(e)$, i.e., the function *g* is a polynomial of degree *s*, it is possible to choose a quadrature rule that is exact for polynomials of degree $k+s$ so the integral in (11) is exact up to machine precision.

There are several virtual element spaces where the virtual function is a polynomial over edges, see for instance [49, 50]. This is also common in the three dimensional space, where the virtual functions are defined as polynomials on the skeleton of the polygon, see, e.g., [45, 51].

##### Remark 3.2

If $s\le k-1$ the integration of (11) is straightforward. Indeed, since the the degrees of freedom of $v_h$ are defined at the Gauß-Lobatto nodes over edges, one can exploit such integration rule and get

12 $$\begin{aligned} {\int _e v_h(t)\,g(t) {\text {d}t} = \sum _{j=0}^{k} \omega _j\,v_h(t_j)\,g(t_j) = \sum _{j=0}^n \omega _j\,\texttt {dof}_{e,j}(v_h)\,g(t_j)\,, } \end{aligned}$$

where $\texttt {dof}_{e,j}(v_h)$ is the dof value at the $j-$th Gauß-Lobatto quadrature point on the edge *e*, while $t_j$ and $w_j$ are the quadrature points and weights, respectively.

##### Remark 3.3

If $\phi _i$ is a virtual basis function associated with the $i-$th Gauß-Lobatto node of the edge *e*, (12) further simplifies to

13 $$\begin{aligned} {\int _e \phi _i(t)\,g(t) {\text {d}t} = \sum _{j=0}^{k} \omega _j\,\phi _i(t_j)\,g(t_j) = \sum _{j=0}^n \omega _j\,\texttt {dof}_{e,j}(\phi _i)\,g(t_j) = \omega _j\,g(t_j)\,.} \end{aligned}$$

Moreover, if the virtual basis function $\phi _i$ is not associated with any Gauß-Lobatto nodes of the edge *e*, or it is associated with a moment D3, such integral is zero and no computations are needed.

##### Coding hint 3.3

In Vem++, degrees of freedom are C++ classes where the most important information about the dofs are stored. Specifically, for the degrees of freedom D1 and D2 the point parameter $t_j$, the weight $\omega _j$ and the identifier of the edge are stored so that the integral of (13) can be computed at once using such data.

***Face integrals and virtual functions***

When you consider a virtual function $v_h$, it is not possible to compute a generic face integral of the form

14 $$\begin{aligned} {\int _E v_h\,g ~\text {d}E\,.} \end{aligned}$$

Indeed, the function $v_h$ is unknown inside the polygon so it can not be evaluated at any quadrature point inside *E*. However, for particular choices of $g(x,\,y)$ it is possible to get the *exact* value of the integral in (14) at once *without* resorting to any quadrature rule since it is exactly a degree of freedom of $v_h$.

Consider for instance the virtual element space defined in Section 2 and the degrees of freedom defined in Section 2.1. If the virtual element approximation is $k=3$, we know the exact result of the following integrals

15 $$\begin{aligned} \int _E v_h~\text {d}E,\qquad \int _E v_h\left( \frac{x-x_E}{h_E}\right) ~\text {d}E\quad \text {and}\quad \int _E v_h\left( \frac{y-y_E}{h_E}\right) ~\text {d}E\,. \end{aligned}$$

Specifically, such integrals are the degrees of freedom of the function $v_h$ and, since we know only the degrees of freedom of the virtual function $v_h$, we know in fact the values of the integrals in (15): we *do not need* any quadrature rule to compute them, we know its exact values for free.

Moreover, if we are considering the basis function associated with such a space, we deduce the values of the integrals in (15) from the type of the dof. If $\phi _i$ is a basis function associated with a dof type $\texttt {D1}$ and $\texttt {D2}$, we have

$$\begin{aligned} \int _E \phi _i~\text {d}E= \int _E \phi _i \left( \frac{x-x_E}{h_E}\right) ~\text {d}E= \int _E \phi _i \left( \frac{y-y_E}{h_E}\right) ~\text {d}E= 0\,, \end{aligned}$$

while, if $\phi _i$ is associated with a dof type $\texttt {D3}$, one of these integral will be 1, see Remark 2.1.

##### Coding hint 3.4

In Vem++ also the $\texttt {D3}$ dofs are a C++ class where it is stored the identifier of the face and the exponent of the monomial it is associated with so that we are able to identify and compute the integrals of (15) at once.

As you can deduce from the above example, the face integrals we are able to compute are related to both the virtual element approximation degree and the virtual element space we are considering. In particular, if we are considering the spaces Section 2 with a VEM approximation degree $k=2$, we are to compute only

$$ \int _E v_h~\text {d}E\,, $$

since we do not have any degrees of freedom associated with the moments

$$ \left( \frac{x-x_E}{h_E}\right) \qquad \text {and}\qquad \left( \frac{y-y_E}{h_E}\right) \,. $$

##### Remark 3.4

Similar consideration can be done for integral over polyhedrons when we are considering a three dimensional setting of the VEM. In this case, we will have some basis functions associated with volume moments so we can have the exact value of them for free [51].

***Integrals related to the computation of*** $~\Pi ^\nabla _{k}$***-projection***

In this section we will give an explicit description on how compute the $\Pi ^\nabla _{k}$ projection although we are dealing with virtual unknown functions. We will focus in the computation of such projection for a generic basis function $\phi _i$, the computation of this operator for a generic virtual function follows similar arguments. We refer the reader to [38] for a more detailed description of this computation. We consider the scaled monomial basis $\{m_j\}_{j=1}^{\pi _k}$ as basis of the polynomial $\Pi ^\nabla _{k}\phi _i$, i.e.,

16 $$\begin{aligned} \Pi ^\nabla _{k}\phi _i = \sum _{j=0}^{\pi _k} c_j\,m_j\,, \end{aligned}$$

where $c_j\in \mathbb {R}$ are the projection coefficients and $\pi _k$ is the dimension of the polynomials of degree lower or equal to *k*. To facilitate explanation, we recall the definition of this projection operator.

17 $$\begin{aligned} \left\{ \begin{array}{rcl} \displaystyle {\int _E} \nabla \Pi ^\nabla _{k}\phi _i\cdot \nabla m_j~\text {d}E& =&  \displaystyle {\int _E} \nabla \phi _i\cdot \nabla m_j~\text {d}E\\[1em] \displaystyle {\int _{\partial E}} \Pi ^\nabla _{k}\phi _i ~\text {d}e& =&  \displaystyle \int _{\partial E}\phi _i ~\text {d}e\end{array} \right. \,. \end{aligned}$$

Notice that here we do not consider a generic polynomial $p_k$. Indeed, we consider the set of $\pi _k$ monomials, $m_j$, that are a basis of $\mathbb {P}_k(E)$ so that we are able to compute the coefficients $c_j$ in (16).

The main issue related to the computation of *all* the quantities in (17) is related to the presence of the virtual function $\phi _i$ at the right-hand sides.

Consider the first condition in (17) and proceed with an integration by parts

18 $$\begin{aligned} \int _E \nabla \phi _i\cdot \nabla m_j~\text {d}E= -\int _E \phi _i\Delta m_j~\text {d}E+ \int _{\partial E} \phi _i\,(\textbf{n}\cdot \nabla m_j)~\text {d}e\,. \end{aligned}$$

Here, the bulk integral is split into two parts: a boundary part and face part. The boundary integral is computable since the virtual function $\phi _i$ is a polynomial on $\partial E$, c.f. “Edge integrals and virtual functions”. Then, the bulk integral is computable too:

$$\begin{aligned} \Delta m_j = \frac{1}{h_E^2}\left( \tilde{c}\,\tilde{m}_j+\overline{c}\,\overline{m}_j\right) \,, \end{aligned}$$

where $\tilde{m}_j$ and $\overline{m}_j$ are scaled monomials of degree $k-2$ and $\tilde{c}$ and $\overline{c}$ are proper constants depending on $m_j$. The integral of (18) is in fact the sum of two integrals where we recognise the definition of moments, (5),

$$\begin{aligned} -\int _E \phi _i\Delta m_j~\text {d}E= -\frac{\tilde{c}}{h_E^2}\int _{E}\phi _i\,\tilde{m}_j~\text {d}E-\frac{\overline{c}}{h_E^2}\int _{E}\phi _i\,\overline{m}_j~\text {d}E\,, \end{aligned}$$

c.f. “Face integrals and virtual functions”. To better understand the previous identity, we show the following example

$$\begin{aligned} -\int _E \phi _i\Delta \left[ \left( \frac{x-x_E}{h_E}\right) ^3 \left( \frac{y-y_E}{h_E}\right) ^2\right] \text {d}E= &   -\frac{6}{h_E^2}\int _{E}\phi _i\,\left( \frac{x-x_E}{h_E}\right) \left( \frac{y-y_E}{h_E}\right) ^2\text {d}E +\\  &   -\frac{2}{h_E^2}\int _{E}\phi _i\,\left( \frac{x-x_E}{h_E}\right) ^3\text {d}E. \end{aligned}$$

##### Coding hint 3.5

From the previous computations, it comes to mind that the VEM is based on monomials and it requires the computation of their derivatives and recognising monomials. As a consequence, in Vem++ we have developed two classes monomial2d and monomial3d come with some basic symbolic calculus facilities. For instance, given two instances of monomial2d, it is possible to get a new monomial2d that is the product between them, see line 6 of Listing 1, or given a monomial2d, it is possible to get its derivatives, see lines 8 and 9 of Listing 1.


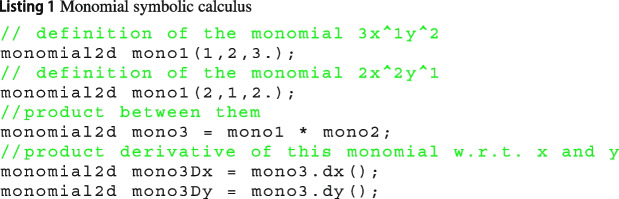


### Analogies with FEM

**Fig. 8:**
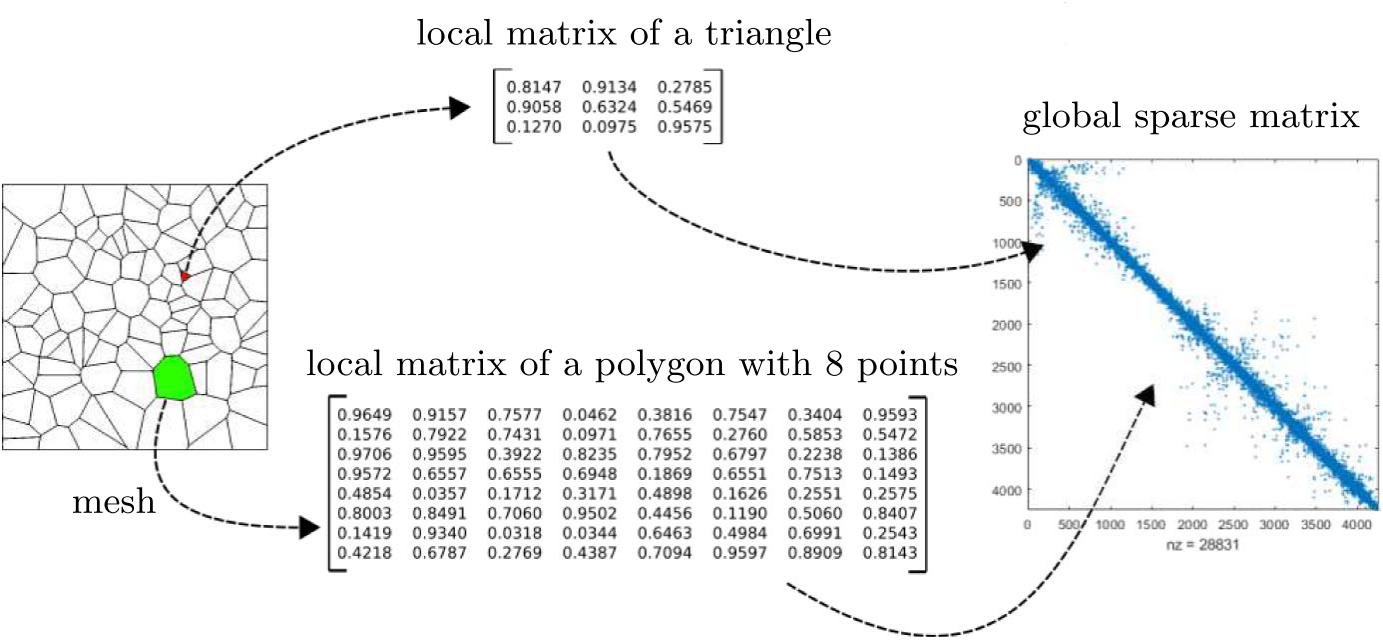
General scheme to find the numerical solution of a PDE, considering a VEM approximation degree $k=1$

The VEM is an extension of the FEM to general polytopal meshes [1]. As a consequence it follows the same workflow of the FEM to assemble and solve the linear system arising from the discretization of a PDE, see Fig. 8:

1. create each local matrices that represent each bilinear or linear forms;
2. assemble such local matrix in the global one;
3. solve the linear system via a proper solver.

The only difference stays in the local matrices. Indeed, in a FEM framework they have always the same size and they are computed starting from the reference element. On the contrary, in the VEM their size depends on the shape of the element and to compute them one needs suitable projection operators. From a more practical point of view, once the code is able to handle such local matrices the implementation of a VEM code and a FEM code coincides.

#### Local matrix definition

Since the main difference between a FEM and a VEM assembling procedure stays in the definition of the local matrices, we focus on how local matrices are handled in Vem++.

Before going into the details, we underline that given a specific linear or bilinear form, it can be seen as a sequence of matrix products and sums. Such practical aspect is well-firmed in the literature, here we are interested in the local matrix data structure implemented in Vem++ so we refer the reader to [38] for more details about that.

For instance, the operator which gives the VEM discretisation of the grad-grad form is

19 $$\begin{aligned} a_h^P(\phi _i,\,\phi _j) = \int _E \nabla \Pi _k^\nabla \phi _i\cdot \nabla \Pi _k^\nabla \phi _i~\text {d}E+ \sum _{r=1}^{\#\text {dofs}}  \texttt {dof}_r\left( \left( I-\Pi _k^\nabla \right) \phi _i\right) \texttt {dof}_r\left( \left( I-\Pi _k^\nabla \right) \phi _j\right) \,. \end{aligned}$$

To achieve this goal, we proceed as follows. We first define the matrices

$$ \textbf{G}_{ij} = \int _E \nabla m_i\cdot \nabla m_j~\text {d}E\qquad \text {and}\qquad \textbf{D}_{ij} = \texttt {dof}_i(m_j)\,, $$

where $\textbf{G}_{ij}$ represents the integral of the inner products between the gradients of the monomial basis, and $\textbf{D}_{ij}$ contains the values of the virtual element degrees of freedom for each monomial basis function. Next, we solve the linear system of (17) for each virtual basis function $\phi _i$ and we collect the solutions in the matrix $\boldsymbol{\Pi }^\nabla _*$. Then, we compute the matrix

$$ \boldsymbol{\Pi }^\nabla =\boldsymbol{\Pi }^\nabla _*\textbf{D}\,, $$

which, by construction, represents the $\Pi ^\nabla _{k}$ operator in the virtual element basis (see Section 3.2 in [38]). Using these matrices, we can compute the form in (19) via the following sequence of matrix products and sums:

20 $$\begin{aligned} \left( \boldsymbol{\Pi }^\nabla _*\right) ^t\,\textbf{G}\,\boldsymbol{\Pi }^\nabla _*+ \left( \textbf{I}-\boldsymbol{\Pi }^\nabla \right) ^t\left( \textbf{I}-\boldsymbol{\Pi }^\nabla \right) \,, \end{aligned}$$

where $\textbf{I}$ is the identity matrix. A more detailed description of this construction can be found in Section 3 of [38].

##### Coding hint 3.6

In this regard we further underline that Remark 3.3 in [38] is particularly important from a coding point of view. Specifically, one can verify the correctness of the projection operators via a proper identity, the so-called “GBD-identity”. This serves as a checkpoint in the debugging process: once GBD-identity is verified, the projection operators *are guaranteed* to be correct. Consequently, if the code produces unexpected results, the issue does not lie in the projection operator. In the folder unitTests/vemMatrix of Vem++, there is a proper GBD-identity test for each projection defined in the library.

From (20) the matrices $\textbf{G},\,\boldsymbol{\Pi }^\nabla _*$ and $\boldsymbol{\Pi }^\nabla$ are essential to compute the local stiffness matrix. However, if we consider a problem with several bilinear forms, such matrices can be also used in the computation of other problem forms. Furthermore, they may also play a key role in some post processing procedure, like the computation of the error. As a consequence, it could be extremely useful to store such matrix and, eventually, load it.

In Vem++, we define a proper data structure that accomplishes all these goals. Such data structure is called vemMatrixHandler. Before describing the implementation detail, we have to spend few words on the “loading process”. In general, a matrix is a collection of numbers arranged in a table. As a consequence, if we store some matrix in a container, it becomes impossible to distinguish them. Consider, for instance, the following matrices

$$ \textbf{H}_{ij}^\nu = \int _E \nu \,m_i\, m_j ~\text {d}E\qquad \text {and}\qquad \textbf{H}_{ij} = \int _E m_i\, m_j~\text {d}E\,, $$

where $\nu$ is a generic function. The matrices $\textbf{H}_{ij}^\nu$ and $\textbf{H}_{ij}$ have the same dimensions, and by simply looking at their coefficients, it is difficult to distinguish between them. In Vem++ we solve this issue in the following way: each matrix is associated with a tag, i.e., a C++ enum variable called matrixType.

The data structure vemMatrixHandler has a “compartment” for each mesh polyhedrons, faces and edges. Then, during the local matrix assembling loops, once a new matrix is computed, it is stored inside a proper container with its tag. As a consequence, during the computation of one specific local matrix, it is possible to access to the compartment and reload the matrices needed. In order to have a clearer idea of the whole procedure, we give an example in Listing 2.
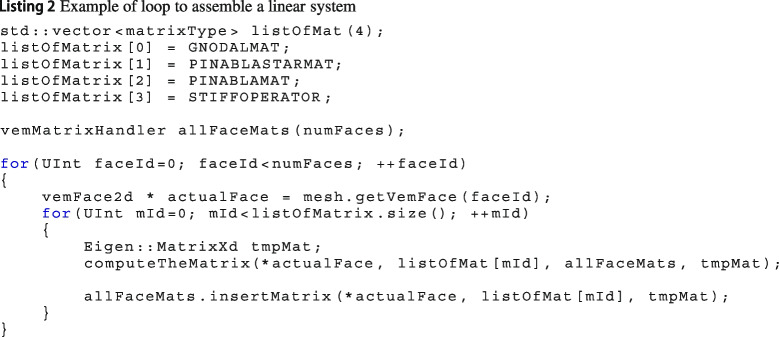


Notice that the function computeTheMatrix, line 15 of Listing 2, takes as input the container itself: the computation may require other matrices that are already inside vemMatrixHandler. From the computation of the local matrix in (20), STIFFOPERATOR, this point becomes more evident. Specifically, it requires the matrices GNODALMAT, PINABLASTARMAT and PINABLAMAT that are the matrices $\textbf{G},\,\boldsymbol{\Pi }^\nabla _*$ and $\boldsymbol{\Pi }^\nabla$, respectively. As a consequence, we are forced to compute local matrices in a precise sequence otherwise we are not able to compute all of them.

On line 17 of Listing 2 we also observe that the local matrix tmpMat is inserted in the container allFaceMats which takes in input: the actual face, actualFace, and its tag, listOfMatrix[matId]. The former information is necessary to put the matrix in the compartment associated with the current face while the latter one is used to uniquely identify it.

## How play with Vem++

As already mentioned in the introduction, the idea behind Vem++ is not to give a code that is used as a black box, but users can “play” with it developing new features and testing their own research. The aim of this section is to further underline this aspect and give the reader more hints about that. In Section 4.1 we show how to define a new local matrix in the code that is the main step to discretise a PDE. Then a user can also define new quadrature formulas and solvers, see Sections 4.2 and 4.3, respectively. Moreover, inside the library there are ten tutorials whose aim is to make the reader understand the code and let him plug in new features.

### New local matrices

In Section 3.2, we have already seen that linear/bilinear forms and projection operators are build via elementary operations among matrices. As a consequence, if users have to develop a new form or projection operator, they have to define a new local matrix. Creating a new matrix in Vem++ is a straightforward procedure and it is deeply described in tutorial 5.

***Define the name of the new local matrix***

Each matrix has a unique tag, i.e., a value of the enum vemMatrixType. To create a new matrix, the first step is defining a new tag, for instance MYNEWMAT, and add it to the vemMatrixType enum, see Listing 3.
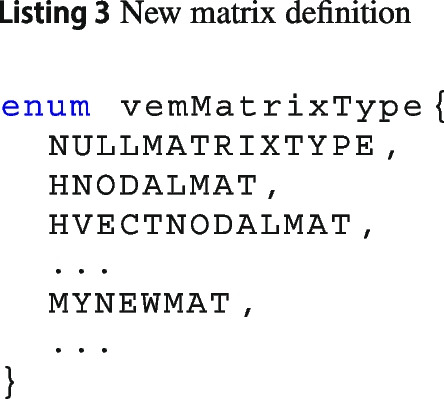


***Define how to compute a matrix***

In Vem++, there is a one-to-one correspondence between C++ enum vemMatrixType and C++ classes. To create a new local matrix, the user has to define a public child of the Vem++ main class vemLocalMatrixDefinition. For instance, in Listing 4, we present the class vem2dMyNewMat that defines a new matrix whose tag is MYNEWMAT. In this implementation, the key point is the definition of the method computeMatrix. This function is a virtual method of the “mother” class vemLocalMatrixDefinition that has to be redefined in the child classes.
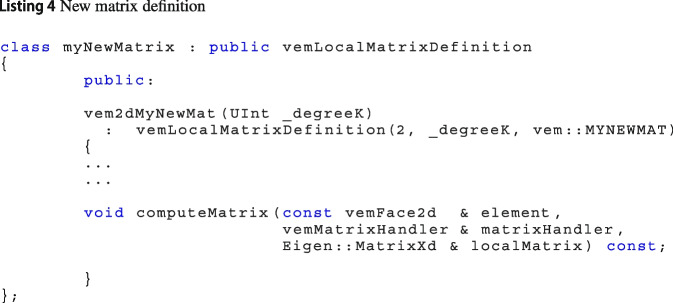


When you are looping over the mesh elements to compute the local matrices the method findOrComputeMatrix of the class vemLocalMatrixDefinition is called, see Linsitngs 5. Such method first looks for the matrix inside the vemMatrixHandler. Then, if it does not find the matrix inside such container, it calls the virtual function computeMatrix whose implementation is in the child class and, as a consequence, the desired matrix is built and then put in the container.
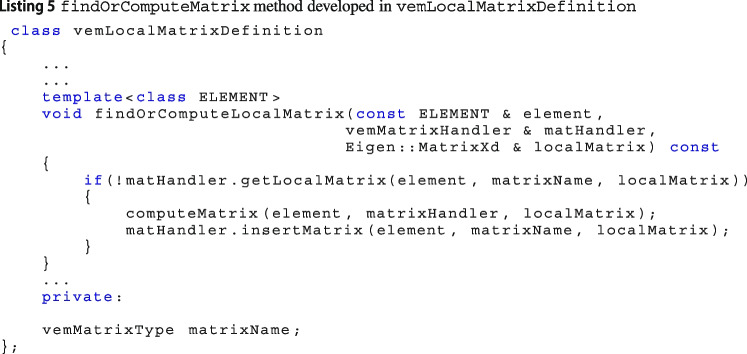


***Add such matrix in the*** ***localAssembler***

The class localAssembler is the factory class that manages the construction of local matrices in Vem++. Once a new matrix is added, the method that computes the matrices in localAssembler has to be properly updated, see Listing 6.
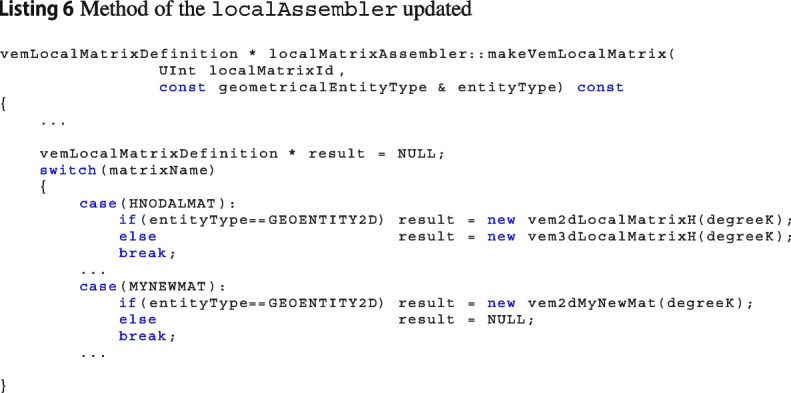


These three steps are required to build a new matrix inside Vem++. In tutorial 5 and tutorial 7, there is a more precise description of this procedure.

### Quadrature rules

One of the challenges in a virtual element code is dealing with the integration over polygons and inside polyhedrons. Independently on the shape of the domain, an integration formula is defined via a set of integration points, $\{\textbf{x}_i\}_{i=1}^n$, and weights, $\{\omega _i\}_{i=1}^n$, i.e.,

21 $$\begin{aligned} \int _P f(\textbf{x})~\text {d}\textbf{x}\approx \sum _{i=1}^n \omega _i f(\textbf{x}_i)\,. \end{aligned}$$

In Vem++ once $\{\textbf{x}_i,\,\omega _i\}_{i=1}^n$ are defined, they are stored inside polyhedrons, polygons or edges, and there is a specific C++ class that will automatically use such list of quadrature points and weights to compute an integral.

For edges, there are already implemented both Gauß-Lobatto and Gauß quadrature formulas. For polygons, a quadrature rule based on sub-triangulation, Vianello’s quadrature rule [46] and the Scaled Boundary Cubature scheme (SBC) [47]. Finally, for polyhedrons, it is implemented one based on sub-tetralisation, an extension of Vianello’s formula to polyhedrons [16] and the SBC scheme for the 3d case.

Moreover, it is also implemented a general rule to make a compression of them [48]. Starting from the set of quadrature points such compression algorithm solve a non negative least squares problem that also ensures that the weights are positive.

Since the integration over polytopes is still an open research field, Vem++ was designed in such a way that adding a new quadrature rule is straightforward. Specifically, if users would like to introduce a new quadrature rule, they have to follow these steps.

***Define the name of the new quadrature formula***

In Vem++ there is an enum used to identify the quadrature points called quadraturePointsType. As a consequence, if you would like to add a new quadrature formula, you have to give it a name, for instance MYQUADFORMULA4PPOLYGONS, and add in such enum, see Linstings 7.
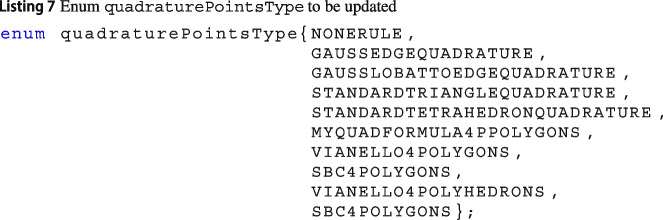


***Define the rule***

There are three main classes to build quadrature rules according to the dimension of the integration domain:

- edgeQuadraturePointsMaker: to compute quadrature rule for edges;
- faceQuadraturePointsMaker: to compute quadrature rule for polygons;
- volumeQuadraturePointsMaker: to compute quadrature rule for polyhedrons.

Each of this class has a method to select which quadrature rule put inside the element. For instance in Listing 8, we show the method to compute the quadrature rule, i.e., the set $\{\textbf{x}_i,\,\omega _i\}_{i=1}^n$ in the case of polygons.
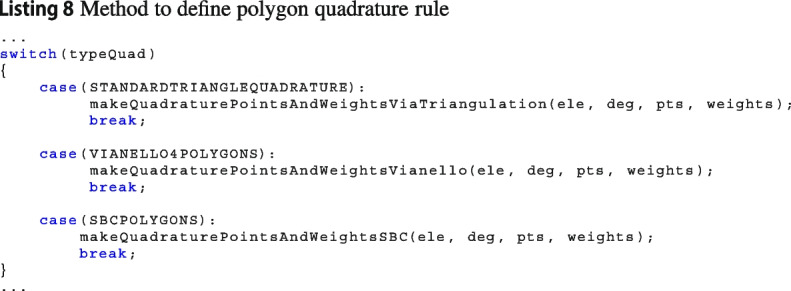
 As a consequence, if users would like to add a new quadrature, they have to enrich such method with the case associated with its quadrature rule, see Listing 9. Such an enrichment can be done adding a proper method in the class faceQuadraturePointsMaker, creating an external class within Vem++ library or via a wrapper of an external library.
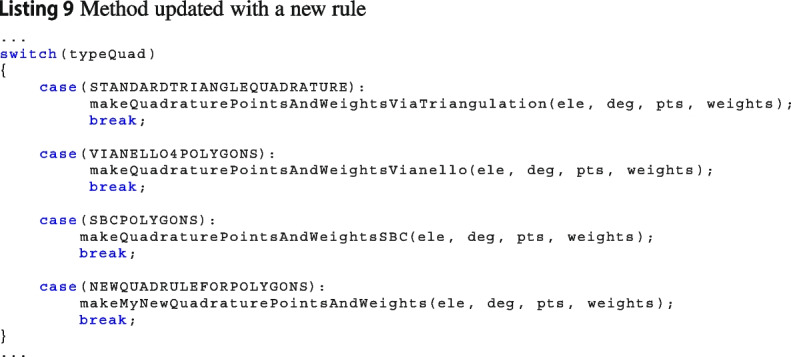


Moreover, inside such class there it is also implemented the compression routine proposed in [48], see the function compressPoints in the class faceQuadraturePointsMaker. As a consequence, once a set $\{\textbf{x}_i,\,\omega _i\}_{i=1}^n$ is built it can be always compressed, i.e., the number of quadrature points can be reduced by properly changing the weights.

### Solvers

Another active research field in the framework of PDEs is finding new ways to solve a linear system arising from the PDE discretisation. Vem++ is designed to facilitate as much as possible researchers interested in such issue.

First, it has already an interface to the most common linear algebra C++ libraries. Specifically, there are two wrapper classes eigenSolver and petscSolver for the C++ libraries Eigen [52] and Petsc [53], respectively. As a consequence, if users have developed a particular solver inside such a libraries, they can look inside those wrappers and call all the routines needed.

Then, since a method to solve a linear system may require additional information about the mesh or the degrees of freedom, in Vem++ there is a class globalSparseSolverData that stores some useful information that are *directly* passed to the solver, see Listing 10

Other than the basic information such as the sparse matrix, matrixSparse, the right hand side, rhs, and the solution vector, solution, This class contains some additional information that can be filled to set particular solvers.

For instance, in Vem++ preconditioners based on field split approach [54] were developed for Maxwell equations [19], the variable listOfFieldData contains all the details about electric and magnetic fields to set the preconditioner. Moreover, when a BDDC preconditioner is set, the method requires the coordinates of pressure dofs [55] and such list of points is stored inside the variable listOfDofData.
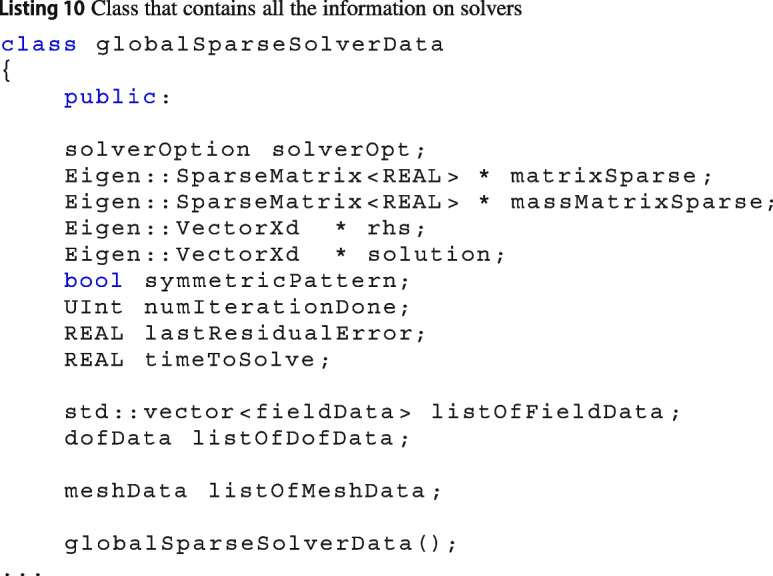


#### Adding a new solver library

The process of adding a new library that solve a linear system is technical but not difficult. The factory class solverHandler is able to distinguish among solvers from different libraries, see Linsting 11. Then, the methods solveWithEigen and solveWithPetsc call the solve method from the wrapper classes of the C++ libraries Eigen and Petsc, respectively.
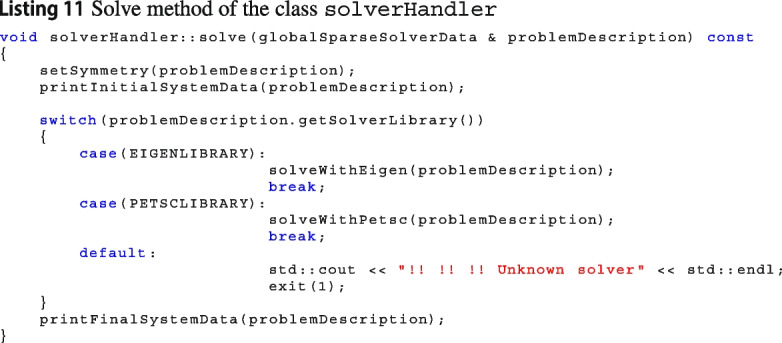


If users would like to add another solver library, they can get ideas on the implementation of the wrappers for Eigen or Petsc.

## Partial differential equations in Vem++

In this section we will show some types of partial differential equation handled by Vem++. Specifically, in Section 5.1, we provide some standard academical problems, while, in Section 5.2, we see some more practical applications where Vem++ was successfully used. For each example, we provide the reference where it was taken so that if readers are interested in such topic, they will have more detail.

### Academic applications

#### Laplacian problems

The first step in the resolution of partial differential equation is the resolution of a Laplacian problem, i.e.,

22 $$\begin{aligned} \left\{ \begin{array}{rll} -\Delta u=& f & \text {in}\,\Omega \\ u=& g & \text {on}\,\partial \Omega \end{array} \right. \,. \end{aligned}$$

In Vem++, it is possible to solve such a problem both in two and three dimensional case and for a generic approximation degree *k*. In [51] it is possible to see all the theory and the results related to such an equation. Moreover, in the library there is the folder bin/ApolloTest that contains all the executables associated with each example of [51].

#### Stokes and Navier-Stokes problems

Vem++ contains also a module related to the resolution of Stokes

23 $$\begin{aligned} \left\{ \begin{array}{rll} {\boldsymbol{\Delta }}\textbf{u}- \nabla p & = {\textbf {f}} & \text {in}\,\Omega \\ \text {div}(\textbf{u})& =0 & \text {in}\,\Omega \\ \textbf{u}& = {\boldsymbol{g}}& \text {on}\,\partial \Omega \end{array} \right. \end{aligned}$$

and the Navier-Stokes problem

24 $$\begin{aligned} \left\{ \begin{array}{rll} {\boldsymbol{\Delta }}\textbf{u}+ \textbf{u}\nabla \textbf{u}- \nabla p & = {\textbf {f}} & \text {in}\,\Omega \\ \text {div}(\textbf{u})& =0 & \text {in}\,\Omega \\ \textbf{u}& = {\boldsymbol{g}}& \text {on}\,\partial \Omega \end{array} \right. \,. \end{aligned}$$

Using the virtual element method to solve such equations is particularly appealing. Indeed, in the VEM it is possible to construct a discrete vector field $u_h$ whose divergence is pointwise zero. This a property is not common to the majority of the finite element approaches and it has a lot of advantages such as the decoupling of the error on velocities and pressure [10]. We refer the reader to [21, 45] to have a deeper analysis on the results obtained by Vem++ for both the two and three dimensional case. Furthermore, if one is interested in the implementation of the code, we refer to the folders bin/Stokes2dPaperTest and bin/Stokes3dPaperTest, where there are the executables associated with the above mentioned papers.

#### Static Maxwell equation in Kikuchi formulation

The virtual element method was successfully applied to solve a magnetostatic problem

25 $$\begin{aligned} \left\{ \begin{array}{rll} {\textbf {curl}}(\textbf{H})& =\textbf{j}& \text {in}\,\Omega \\ \text {div}(\mu \textbf{H})& =0 & \text {in}\,\Omega \\ \textbf{H}\wedge \textbf{n}& ={\boldsymbol{g}} & \text {on}\,\partial \Omega . \end{array} \right. \end{aligned}$$

in the so-called Kikuchi formulation, see, e.g., [56]. More specifically in [57] VEM was successfully applied to general order two dimensional magnetostatic problems. In [58] a lowest order degree for the three dimensional case was defined, while in [59] the general order case for three dimension was introduced. In Vem++ there are all the executables for such papers are in bin/maxwell2d and bin/maxwell3dPaperTest, respectively.

#### Elasticity equation with the Hellinger-Reissner principle

Another interesting problem solved with the VEM is the linear elasticity problems based on the Hellinger-Reissner variational principle. This mixed formulation describes linear elasticity via displacement and the stress fields. Indeed, we solve the following partial differential equation

26 $$\begin{aligned} \left\{ \begin{array}{rll} -\text {div}({\boldsymbol{\sigma }}) & = {\textbf {f}} & \text {in}\,\Omega \\ {\boldsymbol{\sigma }} & = \mathbb {C}\epsilon (\textbf{u}) & \text {in}\,\Omega \\ \textbf{u}& = {\boldsymbol{g}}& \text {on}\,\Gamma _1\\ \boldsymbol{\sigma }\textbf{n}& = {\boldsymbol{\psi }}& \text {on}\,\Gamma _2 \end{array} \right. \,. \end{aligned}$$

where $\textbf{u}$ represents the displacement field and ${\boldsymbol{\sigma }}$ is the stress tensor, $\Gamma _1$ and $\Gamma _2$ are two partitions of the boundary of the domain $\Omega$, where the displacement and the normal component of the stress field are fixed. In a FEM framework, one of the main issue is obtaining a stable and accurate scheme that does preserve both the symmetry of the stress tensor and the continuity of the tractions at the inter-elements. However, we are able to avoid these two drawbacks by exploiting the flexibility of the VEM. For more detail about the mixed linear elasticity based on the Hellinger-Reissner variational principle we refer the reader to [11] and the examples are in the folder bin/HellingerReissnerPaper.

### Practical applications

#### Elasticity with curved edges

In [13], Vem++ was successfully used in two dimensional solid mechanics applications that are characterised by curve boundaries. Specifically they test the virtual element method on two classical benchmark examples involving inelastic materials: the thick-walled viscoelastic cylinder subject to internal pressure and the perforated plastic plate [60].

***The thick-walled viscoelastic cylinder subject to internal pressure***

**Fig. 9:**
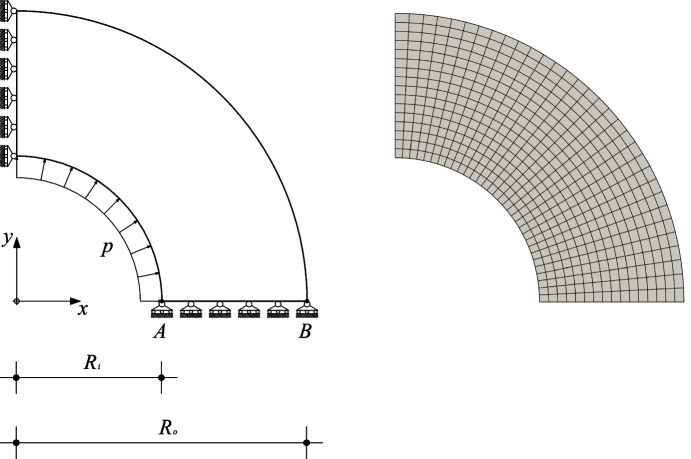
Thick-walled viscoelastic cylinder subject to internal pressure. Geometry, boundary conditions, applied load (left), mesh used (right)

The cylinder has inner [resp. outer] radius $R_i = 2$ [$R_o=4$] and it is subject to uniform pressure *p* on the inner surface, see Fig. 9.

The material is isotropic and it follows a viscoelastic constitutive law as outlined Section 3.1 of reference [61]. We consider two sets of viscoelastic parameters $\left( \mu _0, \mu _1 \right) _{\texttt {v1}} = \left( 0.01, 0.99 \right)$ and $\left( \mu _0, \mu _1 \right) _{\texttt {v2}} = \left( 0.3, 0.7 \right)$, respectively. The former case is devised in a way that the bulk-to-shear moduli ratio for instantaneous loading is given by $K/G(0) = 2.167$ and for long time loading, say at $t=8$, by $K/G(8) = 216.7$, which indicates a nearly incompressible behavior for sustained loading (for instance, at $t = \infty$ the Poisson ratio results 0.498). The second material set indicates an intermediate response at $t = \infty$ after loading is applied. Plane strain assumption is applied in this numerical simulation.

In this test case we make a displacement accuracy analysis between the curved VEM formulation with an approximation degree $k=1,2,3$ and a reference solution. Such reference solution is obtained with quadratic quadrilateral displacement based finite elements with nine nodes *Q*9 running in the FEAP platform using an extremely fine mesh [60].

**Fig. 10:**
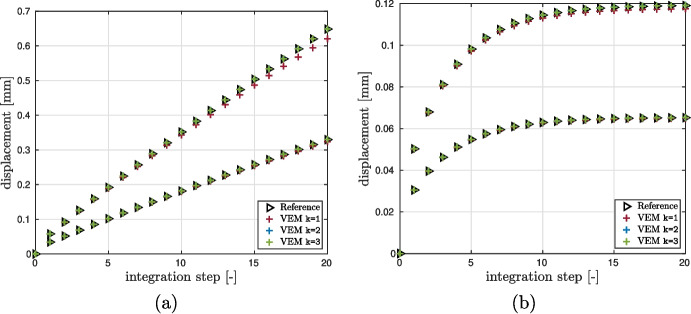
Thick-walled viscoelastic cylinder with internal pressure. Integration step *v*s. radial displacement curves for control points *A* (higher curve), and *B* (lower curve). (a) case $\left( \mu _0, \mu _1 \right) _{\texttt {v1}} = \left( 0.01, 0.99 \right)$; (b) case $\left( \mu _0, \mu _1 \right) _{\texttt {v2}} = \left( 0.3, 0.7 \right)$

The integration-step versus displacement curves for control points *A* and *B*, Fig. 9, is shown in Fig. 10. For both configurations we observe that the solutions provided by Vem++ match the reference one although it decidedly has fewer degrees of freedom.

***The perforated plastic plate***

**Fig. 11:**
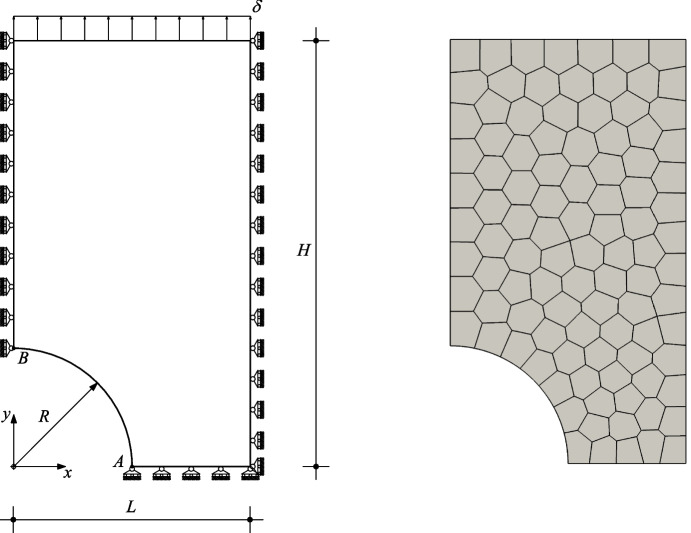
Perforated plastic plate. Geometry, boundary conditions, loading (left), mesh used (right)

In this numerical experiment we consider a rectangular strip with width of $2L = 200$ $\text {mm}$ width and length of $2H = 360$ $\text {mm}$ with a circular hole of $2R = 100$ $\text {mm}$ diameter in its center, see Fig. 11). Material response here follows classical von Mises plastic constitutive model, with material parameters: $E = 7000$ $\text {kg} / \text {mm}^2$, $\nu = 0.3$, and yield stress $\sigma _{\text {y},0} = 24.3$ $\text {kg} / \text {mm}^2$ [60]. We assume plane strain and we use a standard backward Euler scheme with return map projection stress and material moduli computation at the quadrature point level [62]. Displacement boundary restraints are prescribed for normal components on symmetry boundaries and on top and lateral boundaries. Loading is applied by a uniform normal displacement $\delta = 2$ $\text {mm}$ with 400 equal increments on the upper edge, see Fig. 11.

**Fig. 12:**
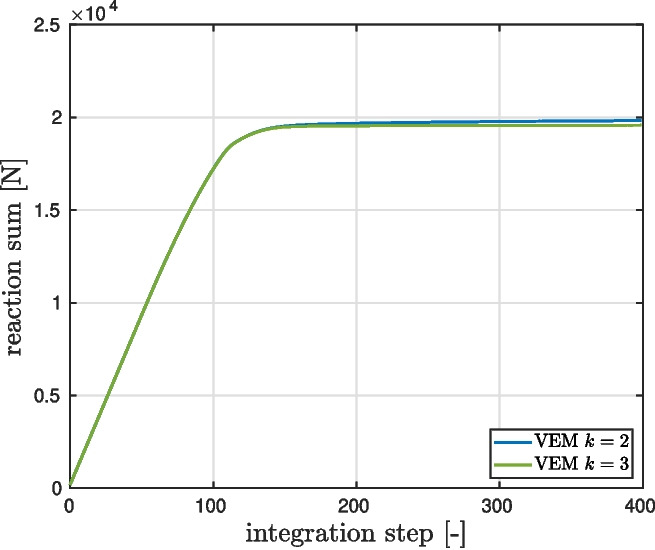
Perforated plastic plate. Structural response computed by Vem++

We proceed with a virtual element simulation via Vem++ and we consider order $k=2,3$ in the curved variant. Accuracy and robustness is assessed by plotting the structural response of the structure, in terms of force reaction sum at the imposed displacement top edge *vs.* integration step, which seems correct for all compared methods and mesh types, showing no significant spurious locking phenomena, see Fig. 12.

#### Magnetostatic

In this subsection Vem++ is tested in applicative magnetostatic problems. We consider a series of numerical experiments and we compare the results obtained via the VEM and a standard FEM. To achieve this goal we take the results provided by MagNet code as a reference solutions [63].

In Vem++ both Kikuchi and the potential formulations are implemented. Then, in the following examples we show the result for each of these approaches and we refer to them as VEM Kik and VEM pot.

***C-core actuator***

In this section we present a numerical simulation of a C-core electromagnet made in Vem++. In this example the electromagnet is composed of a fixed C-shaped core and a movable plunger. A DC current of 1 A supplies the winding around the core limb which in turn excites the magnetic field lines. The overall size of the electromagnet is 80 mm$\times \,$60 mm, while the cross-sectional area of the winding, which incorporates 1000 turns, is equal to 400 mm$^2$.

We focus on the computation of the following physical quantities

1. $B_x$: the *x*-component of the magnetic field $\textbf{B}$ in the mid-point of the air gap;
2. $F_x$: the *x*-component of the force $\textbf{F}$ acting on the plunger, via Maxwell’s stress tensor method,

for different position of the plunger with respect to the C-core. Since we are considering small air-gap widths, a non-linear approximation of the magnetic permeability is required. In the following experiments we define the material of both C-core and plunger as a standard laminated iron with 5 mm thickness [64].

**Fig. 13:**
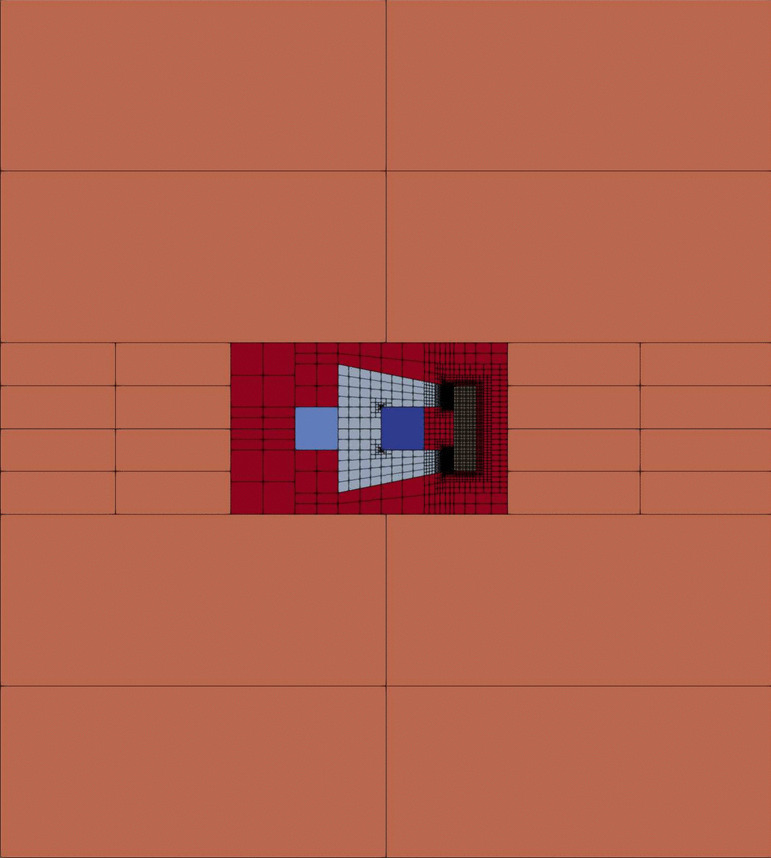
C-core actuator: the whole mesh

Since the physics behind such problem is well known, we can *a priori* identify regions which are particularly interesting from the physical point of view. As a consequence we can generate a mesh refined in the “hot” regions and coarse in the other ones. Such procedure is particularly straightforward with VEM exploiting as much as possible the possibility to add hanging-nodes. Starting from a really coarse mesh, it was possible to generate the mesh represented in Fig. 13.

**Fig. 14:**
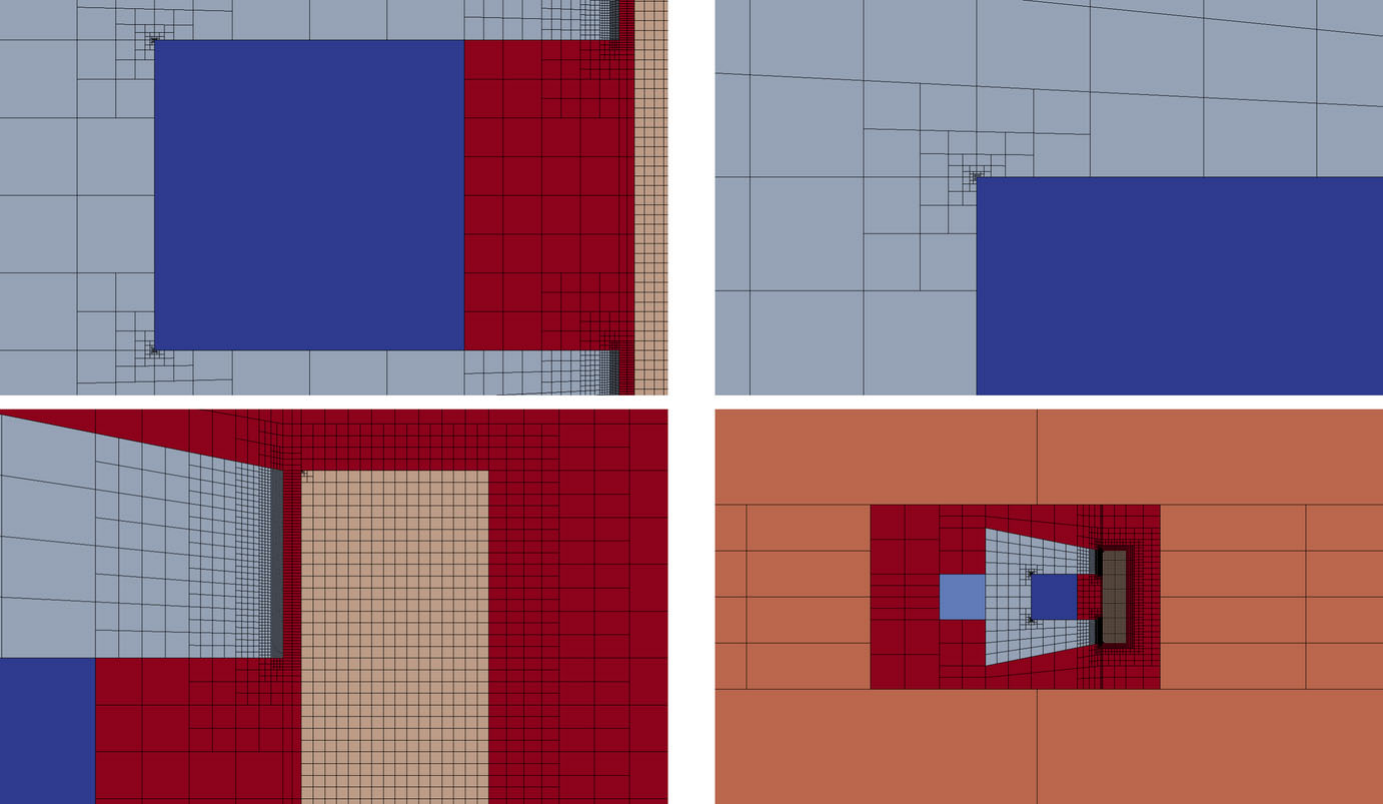
C-core actuator: the whole mesh

Notice that such mesh is refined in the most important regions: at the corners of the C-shaped core where there are singularities of the magnetic permeability; on the boundary of the plunger where there are singularities of the magnetic field and inside the air-gap between the C-core and the plunger, see the details in Fig. 14. Then, other regions where there is no need to have an accurate representation of the solution are not refined and they are charaterised by the presence of a lot of hanging nodes. For instance the mesh inside the two current-carrying regions forming the winding is a really big square element with a lot of hanging nodes, see the details in Fig. 14. Moreover the rectangles far away from the plunger are also big elements that represent the truncated air domain, see the whole domain in Fig. 13 and the details in Fig. 14.

We consider a fixed air-gap of 5 mm, we use the same VEM mesh for both Kikuchi and potential formulation, while we consider a uniform mesh composed of triangles to get a FEM solution via MagNet.

In Table 1 we show the values of $B_x$ and $F_x$ for each method and approximation degree. On the one hand, if we fix the method and we vary the approximation degree, such values becomes stable (the first two digits of both $B_x$ and $F_x$ stay the same). On the other hand if we fix the approximation degree and we vary the method, the computed values are close to each other. This fact is a numerical evidence that the solutions provided by Vem++ are compatible with the one obtained by FEM.

**Table 1:** C-core actuator: $B_x\,$[T] and $F_x\,$[N] computed by FEM and VEM

	VEM Kik	VEM Pot	FEM
Degree	$B_x\,$[T]	$B_x\,$[T]	$B_x\,$[T]
1	0.1225	0.1228	0.1228
2	0.1250	0.1250	0.1227
3	0.1250	0.1250	0.1227
	VEM Kik	VEM Pot	FEM
Degree	$F_x\,$[N]	$F_x\,$[N]	$F_x\,$[N]
1	−5.7325	−5.9803	−5.9898
2	−6.0334	−6.2475	−6.1091
3	−6.0976	−6.2717	−6.1254

Form another point of view we can also state that such results are comparable to a commercial software like MagNet that is one of the best software used in the magnetostatic field.

However, we admit that such comparison is not completely clean. Indeed, the FEM solution is computed on a different mesh, we can not obtain exactly the same values. Moreover, MagNet also makes some post processing on the computed solution to get smooth values of both $B_x$ and $F_x$ and, consequently, its solution converges to different values with respect to ones of Vem++.

Now we proceed with the evaluation of $B_x$ e $F_x$ varying the air-gap width. We compute such solution *only* via Vem++ since we have already validated the method with the previous comparison. Such data are collected in Table 2.

**Table 2:** C-core actuator: values of $B_x$ and $F_x$ by varying air-gap widths

*d* [mm]		0.25	0.50	1.00	2.00
$B_x$ [T]	VEM Kik	2.0428	1.1176	0.5870	0.3015
	VEM Pot	2.0449	1.1185	0.5875	0.3017
$F_x$ [N]	VEM Kik	−1360.5	−413.00	−117.09	−32.498
	VEM Pot	−1365.5	−416.32	−119.26	−33.433

**Fig. 15:**
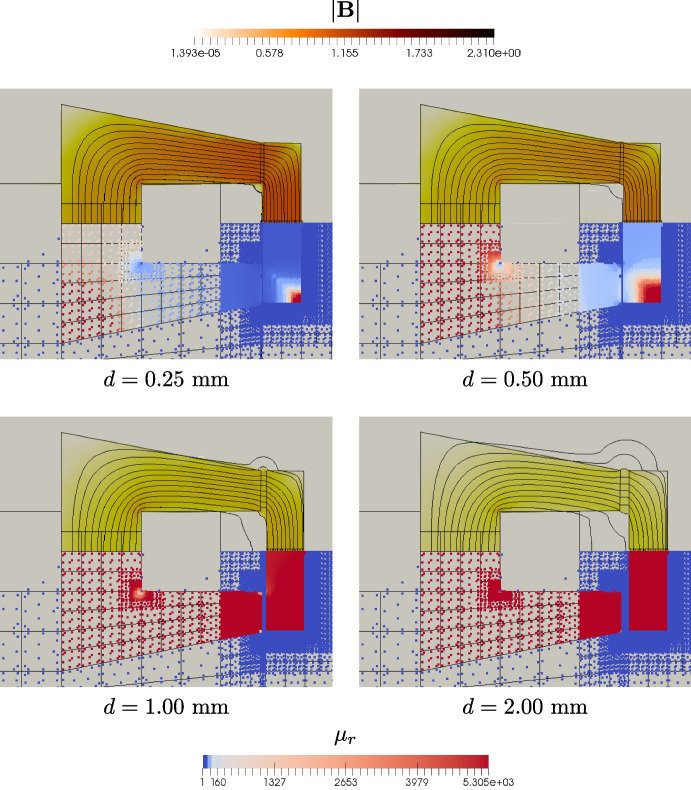
C-core actuator: the vector field $\textbf{B}$, on top of each figure, and the magnetic permeability $\mu _r$ at the quadrature points, on bottom of each figure

Both formulations give similar results by varying the air-gap width, *d*. More specifically, we observe that the trend of $B_x$ and $F_x$ is approximately $d^{-1}$ and $d^{-2}$, respectively, that is perfectly aligned with the theory and the real-life experiments. Finally, in Fig. 15 we show both the vector field $\textbf{B}$ at mesh vertices and the value of $\mu _r$ at the quadrature points.

Such data are computed via the VEM potential formulation (Kik VEM is similar so we do not show it). The behaviour of magnetic field and the magnetic permeability are the expected ones. Indeed, the vectors are properly aligned and the strength of $\textbf{B}$ increases for small air-gap widths. Moreover, both the C-core and the plunger have a uniform $\mu _r$ for large air-gap widths, $d=1.00$ mm and $d=2.00$ mm, while the material starts to saturate when *d* is small.

***Interior permanent magnet motor***

**Fig. 16:**
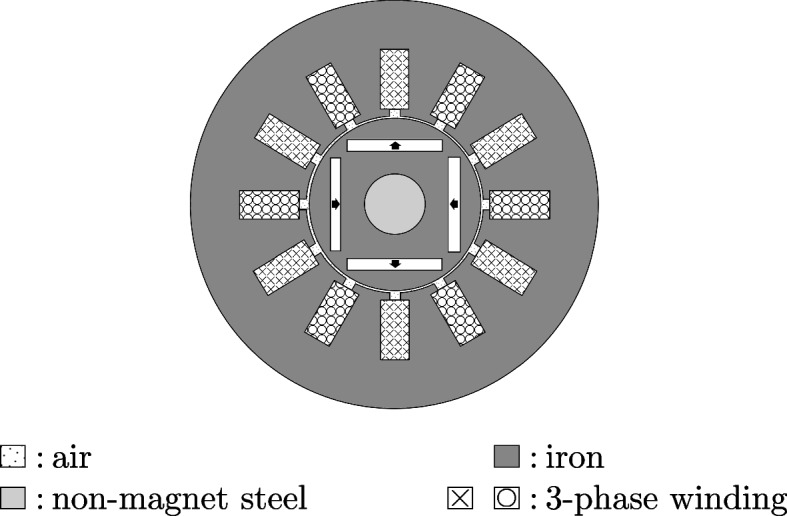
Interior permanent magnet motor: the geometry of the 4-pole motor taken into account

In this section we are using Vem++ to make a numerical simulation of an Interior-Permanent-Magnet (IPM) motor characterized by four poles and 12 stator slots see Fig. 16. The external and the rotor diameters are $68.0~\text {mm}$ and $30.0~\text {mm}$, respectively, while the air-gap width is $0.5~\text {mm}$. The permanent magnet exhibits a radial magnetization with remanent field equal to $\textbf{B}_0=1.~\text {T}$ and coercive field equal to $\textbf{H}_c=7.957\,10^5~\text {Am}^{-1}$.

In this example we are interested in the computation of the cogging torque, i.e., the torque acting on the rotor when the three-phase current in the rotor slots is zero (no-load operation). Such quantity is crucial to design a permanent magnet motor since it takes into account the tendency of the permanent magnet axis to align with the direction that corresponds to the minimum energy stored in the motor.

**Fig. 17:**
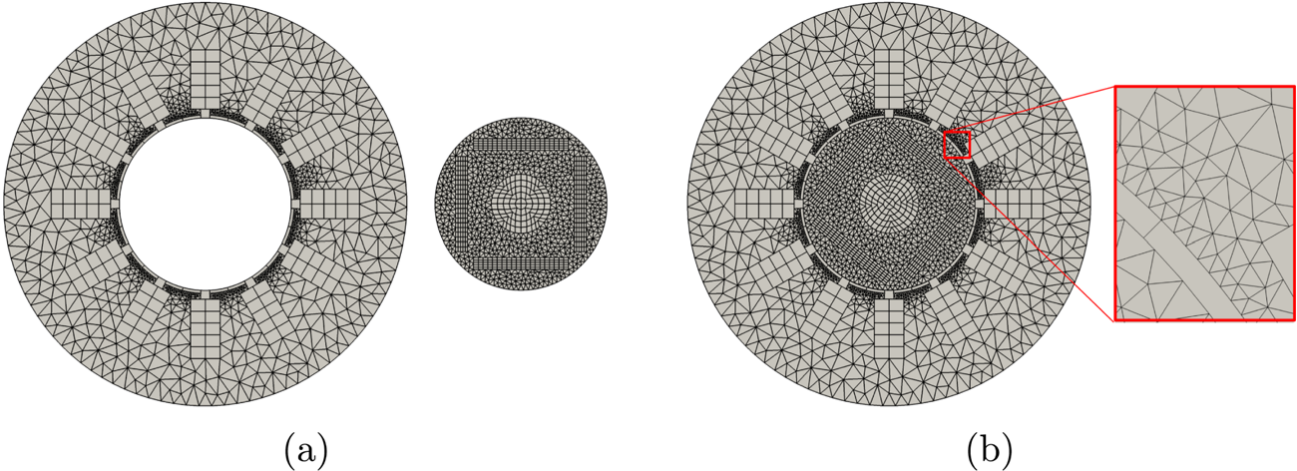
Interior permanent magnet motor: (a) stator and rotor mesh, (b) mesh glued with a detail of the hanging-nodes generated by the gluing procedure

The cogging torque has to be computed considering different angular positions of the rotor. To generate *all* these meshes, we can exploit the flexibility of VEM in gluing meshes. More specifically, we generate stator and rotor mesh separately only one time. Then, to compute the torque cogging torque with different angles, we glue them together with the desired angle to run the computation, see Fig. 17. To achieve this goal in Vem++ it is implemented the class mesh2dMerger. Given two meshes $\Omega _{h,1}$ and $\Omega _{h,2}$ in $\mathbb {R}^2$, such class is able to understand if they have a common boundary and merge them adding hanging nodes, see the detail highlighted in Fig. 17(b). Moreover, as you can see from this example, mesh2dMerger is able to merge two meshes along a curved common boundary and this process is geometry free. Indeed, there is no need of a geometrical description of the curve, mesh2dMerger recognise piece-wise segments on curved boundaries and refine them so that two meshes are joined together, see the detail highlighted in Fig. 17(b).

**Fig. 18:**
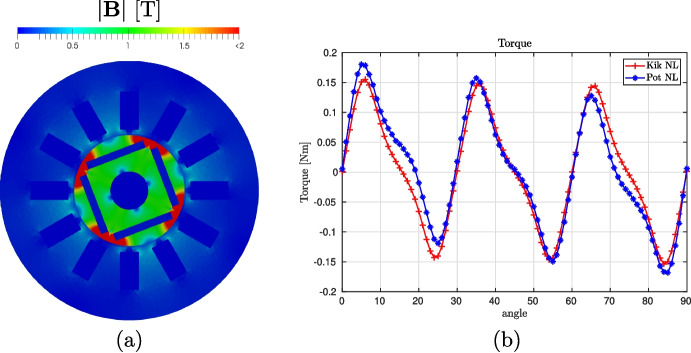
Interior permanent magnet motor: (a) magnetic induction map with an angle of $21^\circ$ (b) values of torque for different positions of the rotor

To compute the cogging torque, we use the Maxwell stress tensor approach considering a cylindrical surface co-axially located with respect to the rotation axis as the integration surface accordingly. In Fig. 18(a) we depict the magnitude of the magnetic field $\textbf{B}$ when the torque-angle is $21^\circ$ degrees while in Fig. 18(b) we show the torque-angle curve for step equal to $1^\circ$. As expected, the torque period is equal to $30^\circ$, indeed

$$ 360^\circ /\text {LCM}(4,12) = 30^\circ \,, $$

where $\text {LCM}$ is the least common multiple operator and 4 and 12 are the numbers of permanent magnets and slots, respectively. Moreover, it exhibits zero mean value over the period. Once again there is a good agreement between Kikuchi and potential virtual element formulations.

**Fig. 19:**
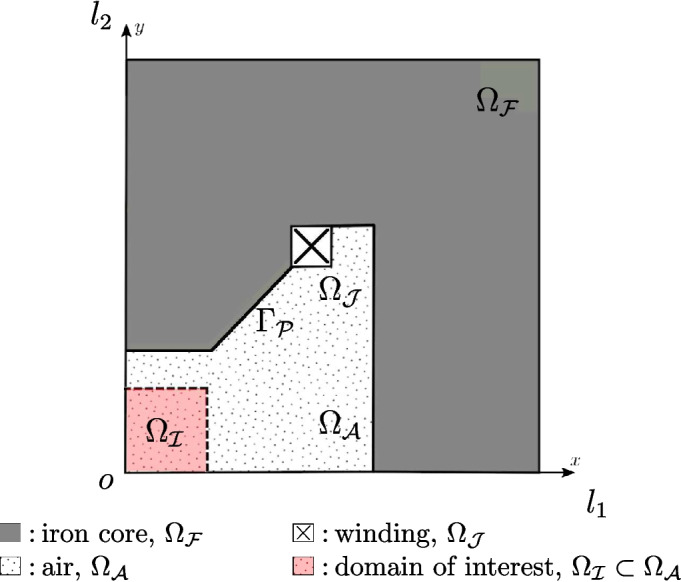
Optimal shape design of an electromagnet: domain to consider

***Optimal shape design of an electromagnet***

In this section Vem++ was used for handling the shape variation of a magnet. More specifically, we consider the optimal design of a magnet for applications in clinical hypethermia.

**Fig. 20:**
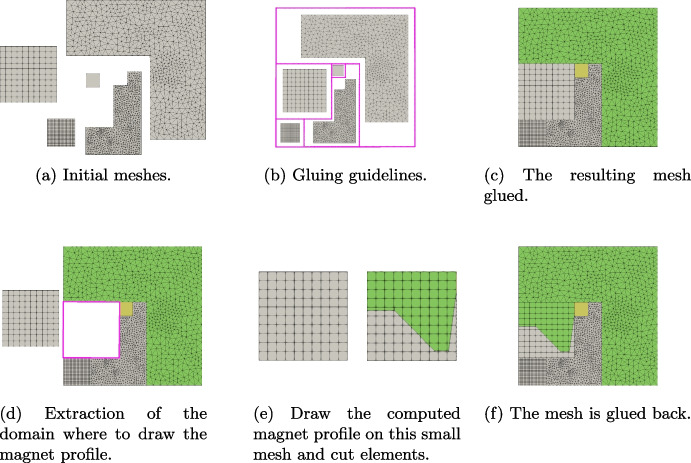
Optimal shape design of an electromagnet: the whole meshing procedure during optimisation

**Fig. 21:**
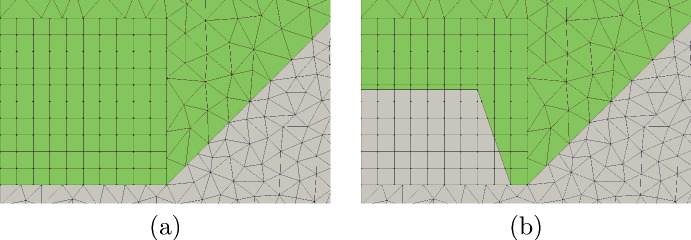
Optimal shape design of an electromagnet: the initial profile (a) and the optimised profile (b)

A typical device is characterised by a magnetic core made of ferrite and it exhibits three limbs. Two series-connected current-carrying coils are wound on the central limb which has a wide air-gap, where the patient is accommodated during the treatment.

A quarter of the model geometry here considered is shown in Fig. 19. A ferrite core fills in region $\Omega _\mathcal {F}$, while an air-gap 30 cm high and 20 cm long incorporates the region of interest $\Omega _{\mathcal {I}}$, i.e., a path along which the degree of uniformity of flux density is controlled. The non-linear $\textbf{B}$-$\textbf{H}$ curve of the ferrite considered in the model exhibits an initial value of relative permeability equal to 1800, and a saturation flux density of 490 mT. The complementary domain includes the winding cross-section $\Omega _\mathcal {J}$ which is composed of 16 turns and carries a sinusoidal current of 150.0 Amps at 100.0 kHz and an air region, $\Omega _\mathcal {A}$.

The design challenge is to shape the magnetic pole, $\Gamma _{\mathcal {P}}$, in such a way that the prescribed field takes place in the region of interest, $\Omega _{\mathcal {I}}$. Such problem is usually reformulated as an inverse problem solved by means of a numerical optimisation technique. Indeed, a suitable functional is defined to measure the discrepancy between actual and the prescribed flux density in the region of interest, $\Omega _{\mathcal {I}}$.

**Fig. 22:**
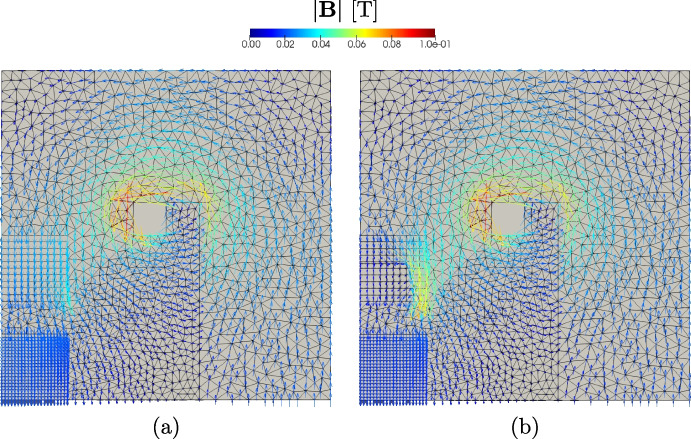
Optimal shape design: field $\textbf{B}$ arrow maps for the designs (a) and (b)

**Fig. 23:**
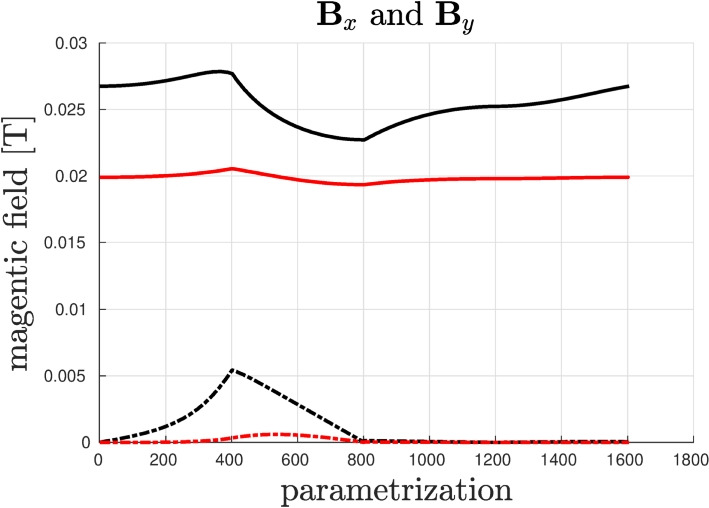
Optimal shape design: comparison between the initial and final values of $\textbf{B}$ in black the values associated with the configuration (a), in red the ones associated with (b)

The proper optimisation procedure is deeply described in [7]. However, in this section we show how to exploit the flexibility in mesh generation of VEM to tackle such procedure. In Fig. 20 we summarise the whole procedure we use to generate the mesh. Each piece of the domain is discretised at the beginning and then glued together using mesh2dMerger, see Fig. 20(a), (b) and (c). Then, the mesh associated with the region where we have to draw the magnet profile is extracted and the structured quadrilateral mesh is stored, see Fig. 20(d). In Fig. 20(e) we show the key step where VEM plays an important role. We start from a uniform mesh composed, e.g. of quadrilateral elements regularly spaced, see Fig. 20(e) (left), and then introduce suitable cuts by segments, see Fig. 20(e) (right). Consequently the original quadrilateral element is cut in a pair of polygons that are naturally handled by VEM and there is no need to sub-triangulate or re-build the mesh. To cut a mesh via affine segments, in Vem++ there is a specific class called addStraightLineOnMesh2d. Finally, this piece of mesh is glued back to the mesh, Fig. 20(f).

Thus, the optimisation procedure looks for the best profile by changing the locations of the points that define it. However, at each optimisation step the mesh is not built from scratch: the meshing part jumps from Fig. 20(e) and (f). As a consequence, the meshing part is quite fast since it has to make a cut of a small part of the domain without touching the other parts and the background mesh is structured.

In Fig. 21(a) we show the initial $\textbf{B}$ computed on the profile depicted in Fig. 22(a), while in Fig. 21(b) the resulting $\textbf{B}$ field on an optimised geometry that is represented in Fig. 22(b).

To have a more qualitative proof of the effectiveness of the optimisation procedure, in Fig. 23 we plot both the *x* and *y* component of the magnetic filed $\textbf{B}$ along the boundary of the domain of interests, $\Omega _{\mathcal {I}}$. If we verify that such components are almost constant on this boundary, the vector field $\textbf{B}$ is also constant inside $\Omega _{\mathcal {I}}$. This fact is due to the presence of homogeneous Neumann and Dirichlet boundary conditions on $y=0$ and $x=0$, respectively, and the fact that there is no current source inside $\Omega _{\mathcal {I}}$. The optimised profile does create a uniform magnetic field inside the region of interest. Indeed the red lines that are associated with the *x* and *y* components of the field $\textbf{B}$ are almost flat.

## Data Availability

No datasets were generated or analysed during the current study.
